# FeCoNiCuPt High‐Entropy Alloy Boosts Photocatalytic Hydrogen Production on Protonated Graphitic Carbon Nitride

**DOI:** 10.1002/advs.74691

**Published:** 2026-03-02

**Authors:** Yunzhu Zang, Jiali Ren, Yanjun Xue, Jian Tian

**Affiliations:** ^1^ State Key Laboratory of Disaster Prevention and Ecology Protection in Open‐pit Coal Mines Shandong Key Laboratory of Special Epoxy Resin School of Materials Science and Engineering Shandong University of Science and Technology Qingdao China

**Keywords:** high‐entropy alloys, photocatalytic hydrogen production, protonated g‐C_3_N_4_ nanosheets, Schottky junction

## Abstract

Among viable approaches to address the current energy crisis, photocatalytic water splitting to produce hydrogen (H_2_) stands out as a promising strategy for converting solar energy into storable chemical energy. In this study, FeCoNiCuPt high‐entropy alloy particles (HEA) are loaded onto protonated g‐C_3_N_4_ nanosheets (HCN NSs) to construct HEA/HCN composites through an electrostatic self‐assembly method. Protonation treatment enriches the surface of g‐C_3_N_4_ nanosheets with abundant active sites and enhances their interfacial charge separation capability. The optimal HEA/HCN composite exhibits a remarkable hydrogen evolution rate of 1672 µmol·h^−1^·g^−1^, representing a 98.35‐fold enhancement compared to pristine HCN. The apparent quantum efficiency of HEA/HCN composite reaches 3.23% at λ = 370 nm. Experimental characterizations reveal that the 2D ultrathin protonated g‐C_3_N_4_ nanosheets possess a substantial specific surface area and shortened charge transfer distance, facilitating rapid migration of photoexcited electrons. The incorporation of HEA cocatalysts not only introduces additional active sites but also establishes Schottky junctions at the HEA/HCN interface. The synergistic effect effectively accelerates electron transport and suppresses the recombination of photogenerated carriers, thereby significantly enhancing the photocatalytic H_2_ production performance. This work provides new insights into the future application of high‐entropy alloys as novel cocatalysts in photocatalysis.

## Graphical abstract

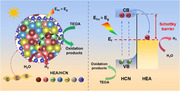

## 1 Introduction

Photocatalytic hydrogen production can utilize abundant solar energy and water resources to generate green, clean, and sustainable hydrogen, which stands as one of the most ideal technologies to address current environmental pollution and the global energy crisis [1, 2, 3]. Over the past decades, significant efforts have been devoted to exploring novel photocatalysts, including metal oxides (TiO_2_) [4], nitrides (g‐C_3_N_4_) [5], sulfides (CdS) [6], and oxyhalides (BiOX, X = Cl, Br, I) [7]. Among these, graphitic carbon nitride nanosheets (g‐C_3_N_4_ NSs) exhibit exceptional chemical stability and represent thermodynamically stable carbon nitride allotrope, with advantages of low cost, facile synthesis, and environmental friendliness [8, 9, 10]. Specifically, lamellar g‐C_3_N_4_ NSs are considered highly promising semiconductor photocatalysts due to their suitable conduction/valence band positions, narrow bandgap, and large specific surface area, enabling widespread applications in photocatalytic hydrogen (H_2_) evolution, nitrogen fixation, CO_2_ conversion, and organic contaminant decomposition [11, 12, 13, 14, 15, 16, 17]. However, the practical photocatalytic hydrogen evolution efficiency of pristine g‐C_3_N_4_ remains severely limited by three intrinsic drawbacks: rapid recombination of photogenerated electron‐hole pairs, restricted visible‐light absorption capability, and insufficient surface‐active sites [18, 19].

To address these limitations and enhance the photocatalytic hydrogen production performance of g‐C_3_N_4_ NSs, researchers have extensively explored strategies including heterostructure construction [20], co‐catalyst loading [21], elemental doping [22], and morphology engineering [23]. In the realm of morphological engineering, strong acid‐protonated g‐C_3_N_4_ (HCN) demonstrates superior attributes compared to pristine g‐C_3_N_4_, including an enlarged specific surface area to furnish abundant surface‐active sites, as well as enhanced charge carrier separation efficiency [24, 25]. Among these approaches, modifying the carbon nitride framework itself (such as by creating intramolecular ternary homojunctions [26] or via C‐ring infiltration to construct S‐scheme homojunctions [27]) remains a significantly effective strategy. However, the synthesis of such crystalline homojunctions requires complex multistep thermal processes, which prompted us to pursue relatively straightforward synthetic approaches. Among these approaches, cocatalyst loading has proven particularly effective for surface modification, as cocatalysts can broaden light absorption, accelerate charge transfer, and suppress photogenerated carrier recombination [28, 29]. Noble‐metal‐containing auxiliary catalysts (including Pt and Au) deposited on g‐C_3_N_4_ NSs demonstrate enhanced performance by both reducing carrier recombination rates and providing abundant active sites [30]. Nevertheless, the prohibitive cost of precious metals severely restricts their industrial scalability [31, 32]. High‐entropy materials have garnered significant attention in recent years for their catalytic potential [33, 34, 35, 36, 37, 38], with their applications rapidly expanding into photocatalytic hydrogen production. Since the first report of high‐entropy oxides for photocatalytic water splitting in 2020 [39], notable progress has been made in this direction. Subsequent studies have explored high‐entropy sulfides, nitrides, and multi‐component alloy systems, which demonstrate considerable advantages in enhancing light absorption, accelerating charge separation, and optimizing surface reaction kinetics, owing to their unique “cocktail effect” and tunable electronic structures [40, 41, 42]. These pioneering works have established high‐entropy materials as a foundational platform for high‐performance and multifunctional photocatalysis. Typically composed of five or more principal elements at near‐equimolar ratios (5–35 at.%), HEAs form thermodynamically stable single‐phase solid‐solution structures [43, 44, 45]. Its core characteristic lies in the high configurational entropy (typically ΔSconf ≥ 1.5R, where R is the gas constant), which serves as a thermodynamic driving force that tends to suppress the formation of intermetallic compounds. Consequently, it stabilizes the formation of a single solid‑solution phase‐such as face‑centered cubic (fcc), body‑centered cubic (bcc), or hexagonal close‑packed (hcp)‐rather than a complex multiphase mixture [46, 47]. The multielement synergy in HEAs enables precise tuning of the catalyst's electronic and band structures, thereby improving photogenerated charge separation/migration and ultimately boosting photocatalytic efficiency [48, 49]. Their high‐entropy effects further stabilize the solid‐solution phase, endowing HEAs with exceptional corrosion resistance, thermal stability, and chemical durability [50, 51, 52]. Through rational elemental design, HEAs can maintain or even enhance photocatalytic performance while minimizing precious metal usage and reducing costs [53]. These advantages make HEAs as highly promising cocatalysts with substantial potential for photocatalytic applications.

Previously reported HEA catalysts largely relied on the extensive use of precious metals. The high cost of precious metals severely limits their industrial‐scale application [54]. Furthermore, their synthesis requires harsh conditions such as high temperature and pressure, making the preparation process difficult and costly [55]. Therefore, the HEA selected in this study ensures excellent catalytic activity while reducing the amount of precious metals used. It also features a simple preparation method and mild reaction conditions that are easy to achieve. In this study, a FeCoNiCuPt high‐entropy alloy (HEA) was rationally designed and synthesized as a co‐catalyst for photocatalytic water splitting. The selection of Fe, Co, Ni, Cu, and Pt follows a deliberate logic: the multiple transition metals (Fe, Co, Ni) aim to modulate the adsorption of reaction intermediates via their incompletely filled d‐orbitals, the introduction of Cu is intended to optimize electrical conductivity, and the incorporation of a small amount of Pt seeks to balance noble metal cost with hydrogen evolution reaction (HER) kinetics. The HEA particles were synthesized via a solvothermal method and subsequently loaded onto protonated g‐C_3_N_4_ nanosheets (HCN) through electrostatic self‐assembly to fabricate HEA/HCN composites. Although the kinetics of the solvothermal process led to a final composition (Fe:Co:Ni:Cu:Pt ≈ 1.4:1.7:1.7:1.9:1) deviating from the ideal equiatomic ratio, all elemental contents remained within the defining range for HEAs (5–35 at.%). Structural characterization by XRD and selected area electron diffraction (SAED) confirmed the formation of a single face‐centered cubic (fcc) solid solution phase, with EDS mapping and line scans demonstrating homogeneous elemental distribution.

The composite with 10 wt.% HEA loading (denoted as HH‐10) exhibited optimal photocatalytic performance, achieving a hydrogen evolution rate of 1672 µmol·h^−1^·g^−1^ under simulated irradiation. This represents a 98.35‐fold enhancement compared to pristine HCN (17 µmol·h^−1^·g^−1^) and significantly outperforms Pt‐based and PtNi‐based reference catalysts. The apparent quantum efficiency (AQE) of the HH‐10 composite reached 3.23% at λ = 370 nm. The exceptional activity is attributed to the synergistic “cocktail effect” of the multi‐elemental HEA and the resulting interfacial optimization, rather than a reliance on strict stoichiometry. Experimental and theoretical analyses elucidate the underlying mechanisms. The successful formation of a Schottky junction at the HEA/HCN interface, driven by favorable work function matching (HEA: 4.54 eV vs. HCN: 3.06 eV), was crucial for enhancing charge separation. This is corroborated by the significantly enhanced photocurrent response and reduced electrochemical impedance observed for the composite. Furthermore, density functional theory (DFT) calculations revealed that the multi‐elemental interface synergistically optimizes the hydrogen adsorption free energy (ΔG_H*_) at various metal sites, bringing them close to the ideal value and thereby providing a thermodynamic foundation for an efficient HER pathway. In summary, this interfacial modification collectively increases the number of active sites, accelerates electron transport, and suppresses charge carrier recombination, leading to a remarkable improvement in photocatalytic hydrogen production performance.

## 2 Results and Discussion

**SCHEME 1:**
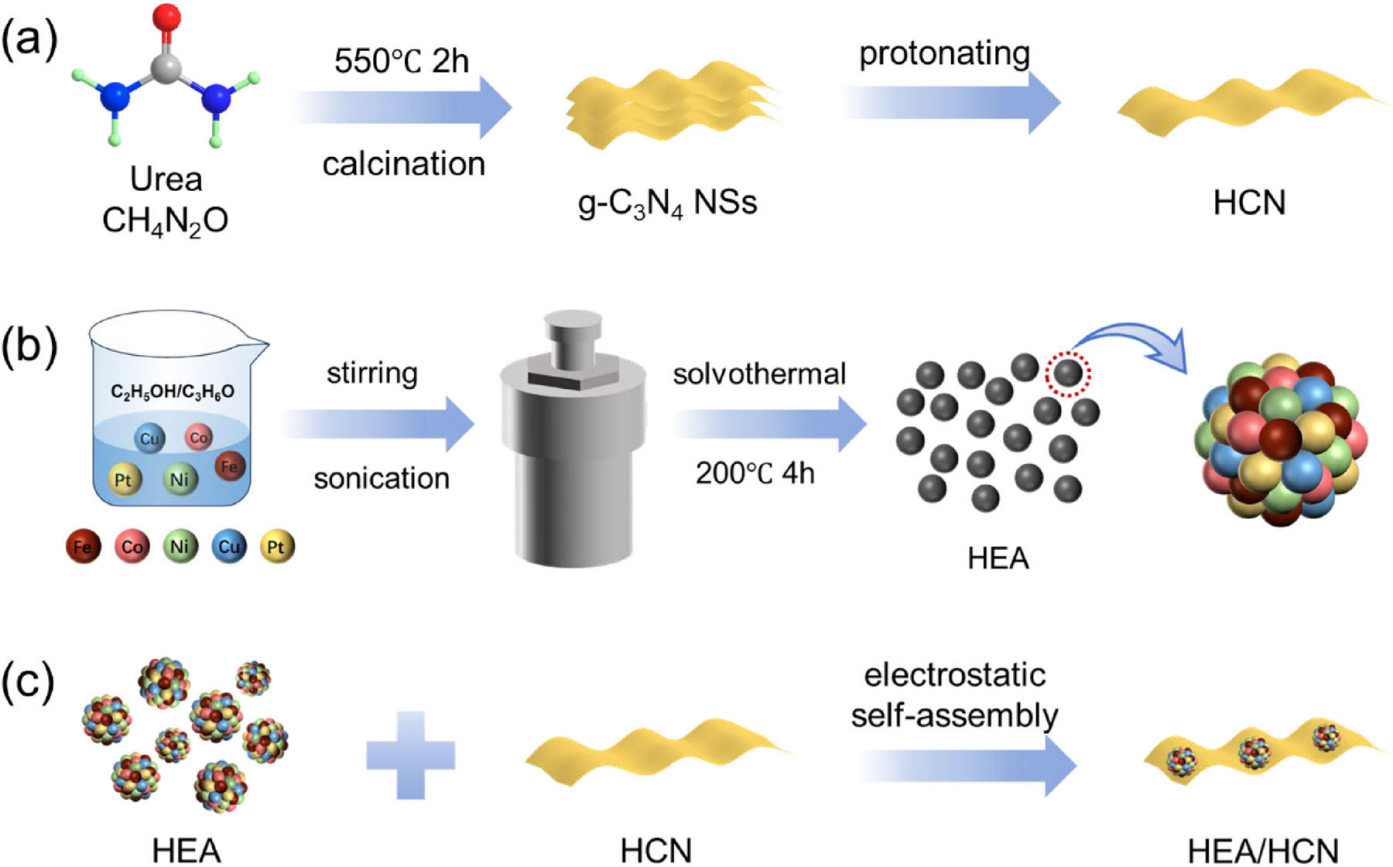
Schematic representation of the synthesis of (a) HCN, (b) HEA, and (c) HEA/HCN.

Graphitic carbon nitride nanosheets (g‐C_3_N_4_ NSs) were synthesized via pyrolytic polymerization of urea precursor, subsequently succeeded by protonation to obtain HCN (Scheme 1a). High‐entropy alloy (HEA) particles were fabricated through a solvothermal method using acetylacetonate precursors (Scheme 1b). The HEA/HCN composite was subsequently prepared via electrostatic self‐assembly method (Scheme 1c). As shown in Figure S1, the zeta potential measurements performed in water at pH 7 yielded values of +14.8 mV for HEA and ‐10.4 mV for HCN. This significant charge polarity establishes an electrostatic driving force that enables the formation of HEA/HCN heterostructures through self‐assembly.

**FIGURE 1:**
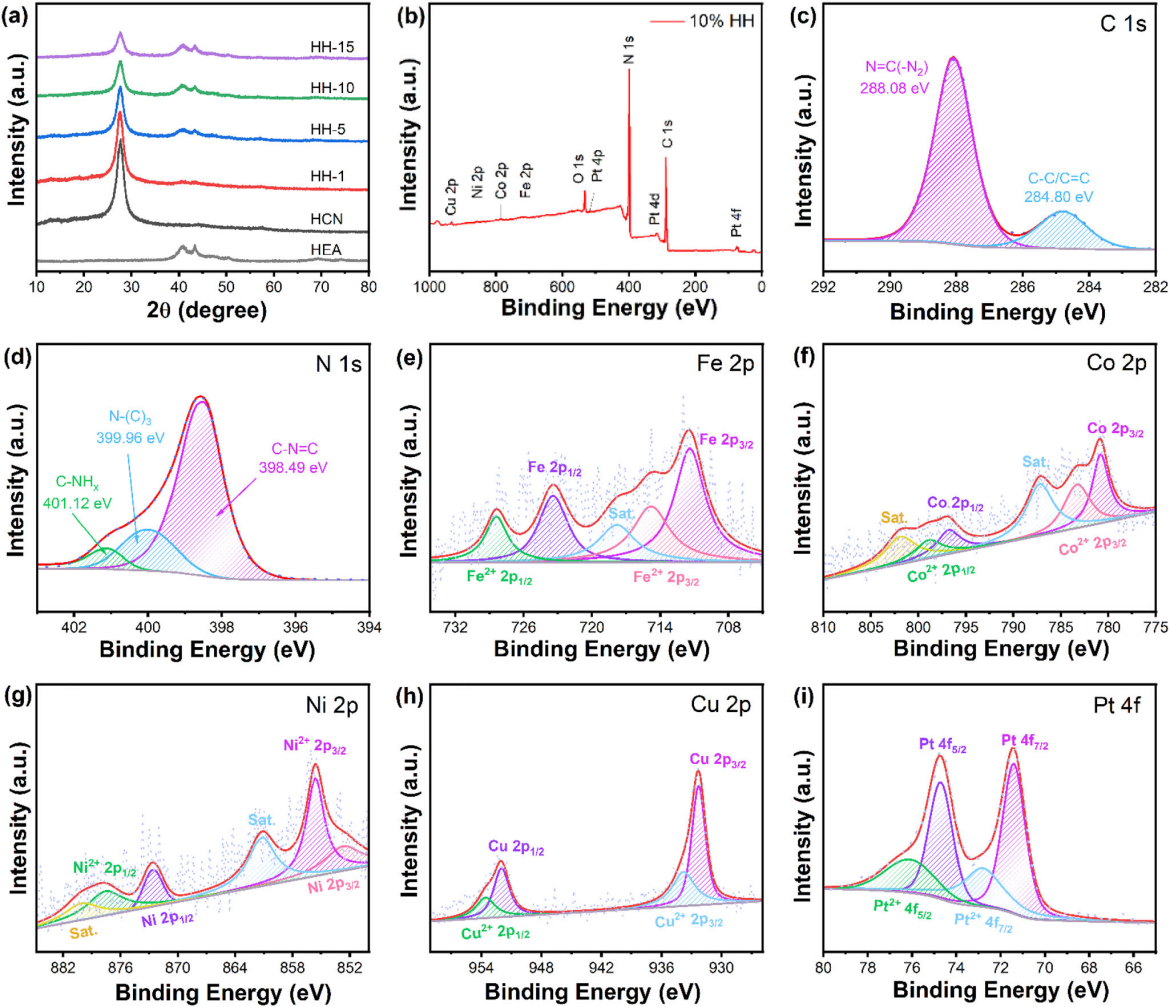
(a) XRD spectra of HEA, HCN and different ratios of HH‐x composites (x = 1, 5, 10, 15); (b) Survey, (c) C 1s, (d) N 1s, (e) Fe 2p, (f) Co 2p, (g) Ni 2p, (h) Cu 2p and (i) Pt 4f XPS spectra of HH‐10.

The crystal structures and phase compositions of the synthesized HEA, HCN, and HH‐x composites were analyzed by X‐ray diffraction (XRD), with results presented in Figure 1a. The XRD pattern in Figure S2 confirms that the HEA particles crystallize in a face‐centered cubic (fcc) structure, characterized by two distinct diffraction peaks at 41.1° and 44.8°, which correspond to the (111) and (200) crystallographic planes, respectively (PDF card JCPDS 48–1549) [56, 57, 58]. This observed lattice alignment is consistent with the well‐documented phenomenon in multi‐component high‐entropy alloys, where the lattice parameter inherently varies with composition‐a fundamental characteristic of such systems [59, 60]. This intrinsic compositional tunability of the lattice provides a crucial microstructural parameter for tailoring material properties. For HCN, two distinct diffraction peaks are observed at 13.1° and 27.3°, indexed to the (100) and (002) planes of graphitic carbon nitride (PDF card JCPDS 87–1526). The diffraction peak at 13.1° corresponds to the planar periodic arrangements of tri‐s‐triazine motifs within the (100) crystallographic plane, whereas the 27.3° reflection is indicative of stacking interactions between aromatic lamellae along the (002) orientation. The presence of these two characteristic peaks confirms the successful synthesis of HCN. Notably, comparative analysis demonstrates a systematic enhancement of HEA diffraction intensities and concurrent attenuation of HCN peaks in HH‐x composites as the HEA loading increases from 1 to 15 wt.% (Figure 1a). This progressive intensity modulation with composition variation provides conclusive evidence for successful HEA deposition on HCN substrates. FT‐IR spectroscopy further confirms the successful synthesis of HCN. As shown in Figure S3, both HCN and the HH‐10 composite exhibit characteristic bands of graphitic carbon nitride at 809, 1200–1700, and 3000–3400 cm^−^^1^. The broad absorption at 3000–3400 cm^−^^1^ corresponds to O‐H/N‐H stretching vibrations. Peaks observed between 1200 and 1700 cm^−^^1^ can be ascribed to stretching modes of C‐N heterocycles in the carbon nitride framework, while the distinct band at 809 cm^−^^1^ is characteristic of triazine ring units in g‐C_3_N_4_ [61, 62, 63, 64]. FT‐IR results indicate that the introduction of HEA did not alter the bulk chemical structure of g‐C_3_N_4_. However, subtle changes in certain spectral regions suggest the presence of interfacial interactions between the metal and the support. These interactions are primarily due to electron transfer driven by the work function difference (such as in a Schottky junction) and physical contact from electrostatic assembly, rather than the formation of strong chemical bonds. ICP‐OES analysis of HEA and HH‐10 is conducted to determine elemental compositions of Fe, Co, Ni, Cu, and Pt. As summarized in Table S1, the atomic ratios in HEA are calculated as Fe:Co:Ni:Cu:Pt ≈ 1.4:1.7:1.7:1.9:1. These ratios further demonstrate that the five‐component composition, with each element's content falling within the 5–35 at.% range, satisfies the concentration criterion defined for high‐entropy alloys, and the single‐phase solid solution observed by XRD justifies the classification of the FeCoNiCuPt material as a high‐entropy alloy (HEA). For the HH‐10 composite, corresponding ratios of ≈1.7:1.8:1.8:1.9:1 are obtained, with the sum of mass percentages for these five elements reaching approximately 9.4 wt.%. This value corresponds to the theoretical HEA loading in the composite material.

The surface chemical states and elemental composition of HH‐10 composite materials were systematically characterized through X‐ray photoelectron spectroscopy (XPS), with the corresponding spectra displayed in Figure 1b–i. All binding energies are referenced to the C 1s peak at 284.80 eV. The survey spectrum (Figure 1b) confirms the presence of C, N, Fe, Co, Ni, Cu, and Pt in HH‐10 composites, verifying the successful incorporation of HEA particles on protonated g‐C_3_N_4_ nanosheets [57]. The observed O 1s signal is attributed to surface oxidation and adsorbed species [65]. Deconvolution of the C 1s XPS profile (Figure 1c) reveals two characteristic binding energy signatures centered at 284.80 and 288.08 eV, which are respectively assigned to C─C/C═C and N═C‐N_2_ moieties within protonated g‐C_3_N_4_ nanosheets [66]. In the N 1s spectrum (Figure 1d), three resolved peaks were identified at 398.49 eV (C─N═C), 399.96 eV (N‐(C)_3_), and 401.12 eV (C‐NH_x_), consistent with the characteristic nitrogen bonding configurations of protonated g‐C_3_N_4_ nanosheets [67]. The Fe 2p spectrum (Figure 1e) exhibits spin‐orbit splitting with peaks at 711.38 eV (Fe 2p_3/2_) and 723.48 eV (Fe 2p_1/2_), while the Co 2p spectrum (Figure 1f) shows characteristic doublets at 780.88 eV (Co 2p_3/2_) and 797.10 eV (Co 2p_1/2_). The Ni 2p spectrum (Figure 1g) reveals coexistence of metallic Ni^0^ (852.68 eV) and oxidized Ni^2+^ (855.68 eV), accompanied by a satellite peak at 861.08 eV. The predominance of Ni^2+^ over Ni^0^ reflects nickel's high chemical reactivity [68]. Similarly, the Cu 2p spectrum (Figure 1h) demonstrates mixed oxidation states with Cu^0^ (932.07 eV) and Cu^2+^ (933.78 eV). The Pt 4f spectrum (Figure 1i) features dominant Pt^0^ species at 71.38 eV (Pt 4f_7/2_) and 74.70 eV (Pt 4f_5/2_), with minor Pt^2+^ contributions [69]. This comprehensive XPS analysis conclusively confirms the successful synthesis of HEA/HCN composites through HEA deposition on HCN substrates. The metallic oxidation state signals observed in the XPS spectra primarily originate from slight surface oxidation of the nanoparticles, while their bulk structure is confirmed by XRD to be a face‐centered cubic (fcc) solid solution alloy.

**FIGURE 2:**
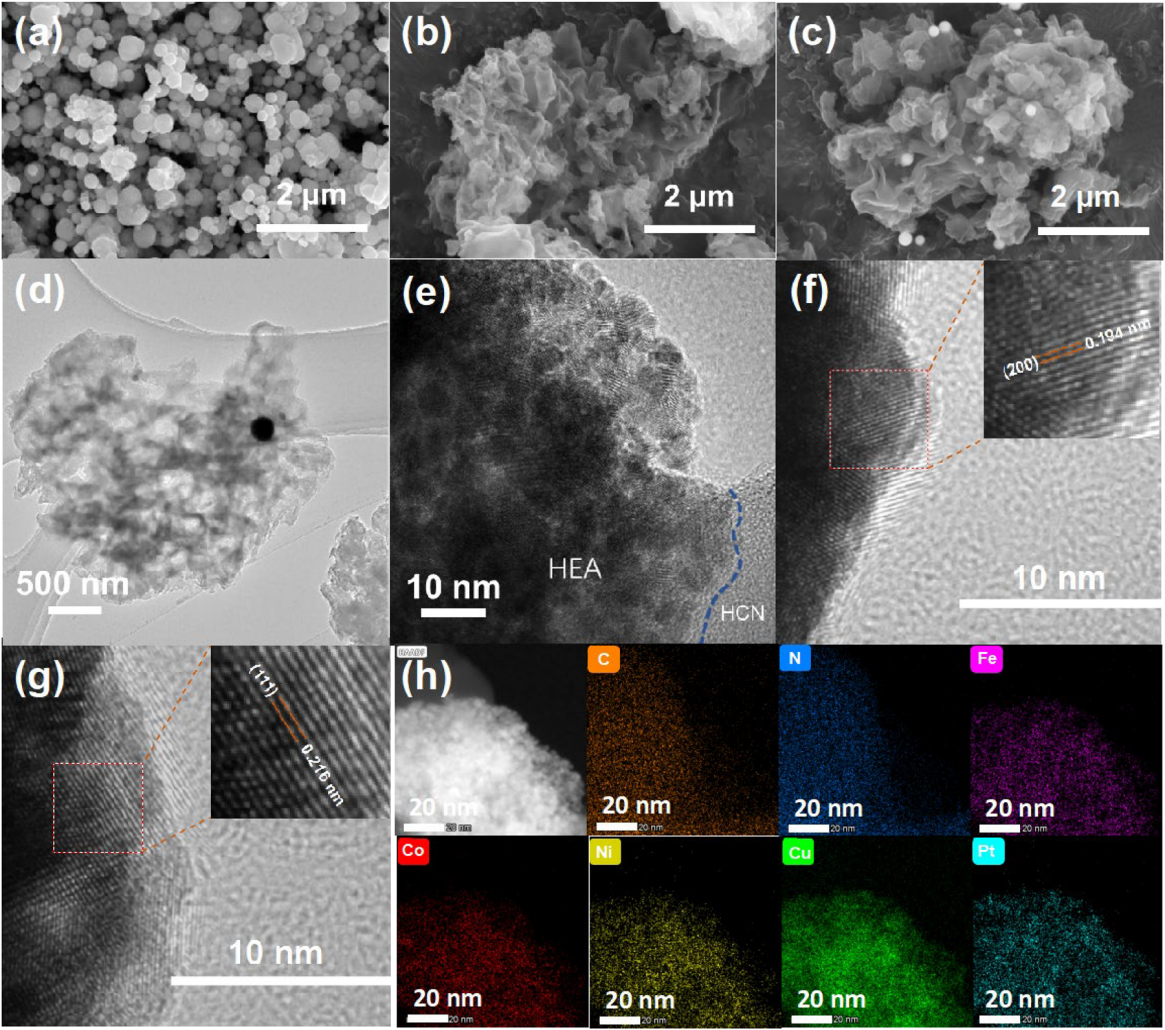
SEM images of (a) HEA and (b) HCN; (c) SEM, (d–g) TEM, and (h) EDS elemental mapping images of HH‐10 composite.

The morphology and microstructure of HH‐10 composites were systematically characterized using SEM, TEM, and EDS to validate the successful loading of high‐entropy alloy (HEA) particles onto protonated g‐C_3_N_4_ substrates, as shown in Figure 2. The synthesized HEA particles exhibit diameters of approximately 50–180 nm (Figures 2a; S4). The SEM image of HCN (Figure 2b) reveals wrinkled nanosheets with irregularly curled edges and lamellar structure that provides a high specific surface area. This morphology enhances water adsorption capacity, offers abundant growth sites for HEA particles, and facilitates the formation of catalytically active sites, thereby contributing to improved photocatalytic performance. Figures 2c,d and S5 demonstrate that HEA/HCN composites retain a 2D porous lamellar architecture with multilayered stacking. HEA particles are uniformly dispersed on ultrathin protonated g‐C_3_N_4_ nanosheets, establishing intimate interfacial contacts that promote photogenerated electron migration and surface reactions. The high‐resolution TEM (HRTEM) image (Figure 2e) confirms the well‐defined HEA/HCN interface, though no lattice fringes are observed for protonated g‐C_3_N_4_ due to its low crystallinity. Fast Fourier Transform (FFT) was employed to measure the interplanar spacing (Figure S6). In the HEA/HCN composite (Figure 2f), the measured interplanar spacing of 0.194 nm matches the (200) crystallographic plane in HEA [70]. Additional lattice fringes in Figure 2g display a spacing of 0.216 nm, matching the (111) plane of HEA [48, 71], consistent with XRD observations. These findings confirm the formation of coherent interfacial contact between HCN and HEA. Figure S7 presents the SAED pattern of the HH‐10 composite, in which the crystallographic planes corresponding to the (111) and (200) reflections of the fcc structure were observed. EDS elemental mapping images (Figure 2h) confirm the homogeneous distribution of C, N, Fe, Co, Ni, Cu, and Pt throughout the HH‐10 composite, providing conclusive evidence for the successful fabrication of HEA‐loaded HCN NSs. Further evidence for homogeneous elemental distribution was obtained from HAADF‐STEM and EDS line‐scan analysis of HH‐10 (Figure S8). The line‐scan profile across an individual HEA particle shows that all five metallic elements exhibit perfectly synchronized signal trends. The superimposed Cu signal from the TEM grid intensifies and diminishes in unison with the signals of the other four elements, confirming their simultaneous presence. Thus, the HEA particles in the HH‐10 composite show no indication of elemental segregation or a core‐shell structure.

**FIGURE 3:**
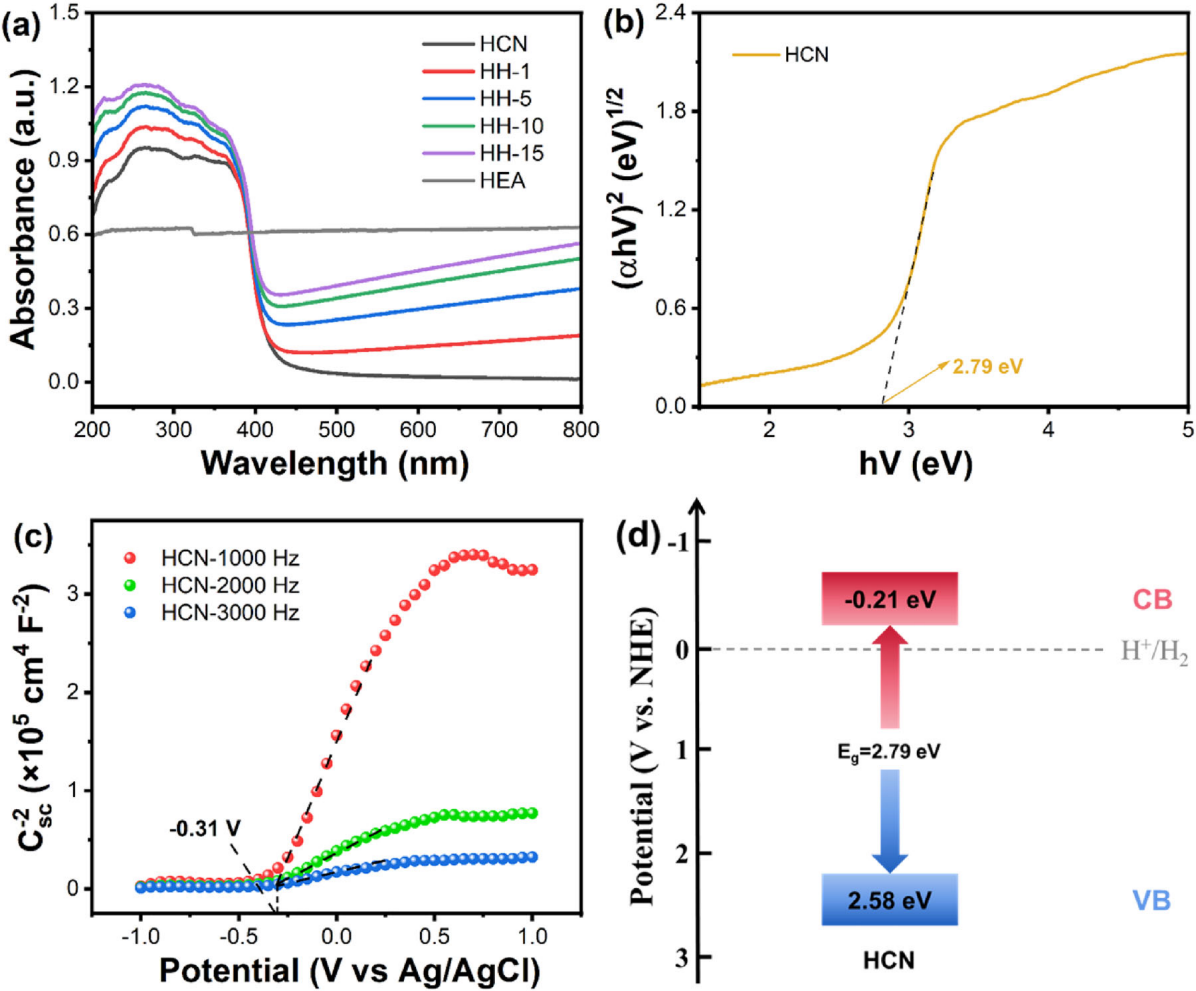
(a) UV–vis absorption spectra of HEA, HCN, and HH‐x (x = 1, 5, 10, 15) composites; (b) Bandgap value, (c) Mott–Schottky diagram, and (d) schematic energy band structure of HCN.

The light absorption properties of HEA, HCN, and HH‐x composites were systematically investigated using ultraviolet‐visible diffuse reflectance spectroscopy (UV–vis DRS). As shown in Figure 3a, the light absorption capacity and spectral range of HH‐x composites progressively enhance with the increase of HEA loading. At the maximum loading of 15 wt.%, the HH‐15 photocatalyst exhibited the strongest absorption intensity, with its absorption edge extending from 420 nm (pristine HCN) to 447 nm. Compared to pure HCN, the introduction of HEA cocatalyst enabled carrier excitation at lower energy thresholds, significantly improving photon utilization efficiency and elevating photogenerated carrier concentrations in the composites. This optimization facilitates the participation of more photogenerated electrons during the light‐driven water dissociation process for hydrogen production, thereby enhancing overall photocatalytic activity.

The bandgap energy (E_g_) of HCN was calculated to be approximately 2.79 eV employing Tauc's extrapolation method based on the (αhν)^1/2^ ∝ hν—E_g_, as shown in Figure 3b. Mott‐Schottky measurement is performed on HCN to estimate its conduction band (CB) potential. The positive slope of the Mott‐Schottky plot (Figure 3c) confirms the n‐type semiconductor behavior of HCN, with a flat band potential (E_FB_) of −0.31 V vs. Ag/AgCl, corresponding to −0.11 V vs. the normal hydrogen electrode (NHE). For n‐type semiconductors, the conduction band potential (E_CB_) is typically ∼0.1 V lower than E_FB_, yielding an E_CB_ of −0.21 V vs. NHE for HCN [30]. Combined with the bandgap value (2.79 eV, Figure 3b), the valence band potential (E_VB_) of HCN is determined as 2.58 V vs. NHE. The derived energy band structure of HCN relative to the standard hydrogen electrode is illustrated in Figure 3d. The conduction band potential of HCN (−0.21 V vs. NHE) is more negative than the H^+^/H_2_ redox potential (0 V vs. NHE), confirming its thermodynamic capability for photocatalytic water splitting toward hydrogen evolution.

**FIGURE 4:**
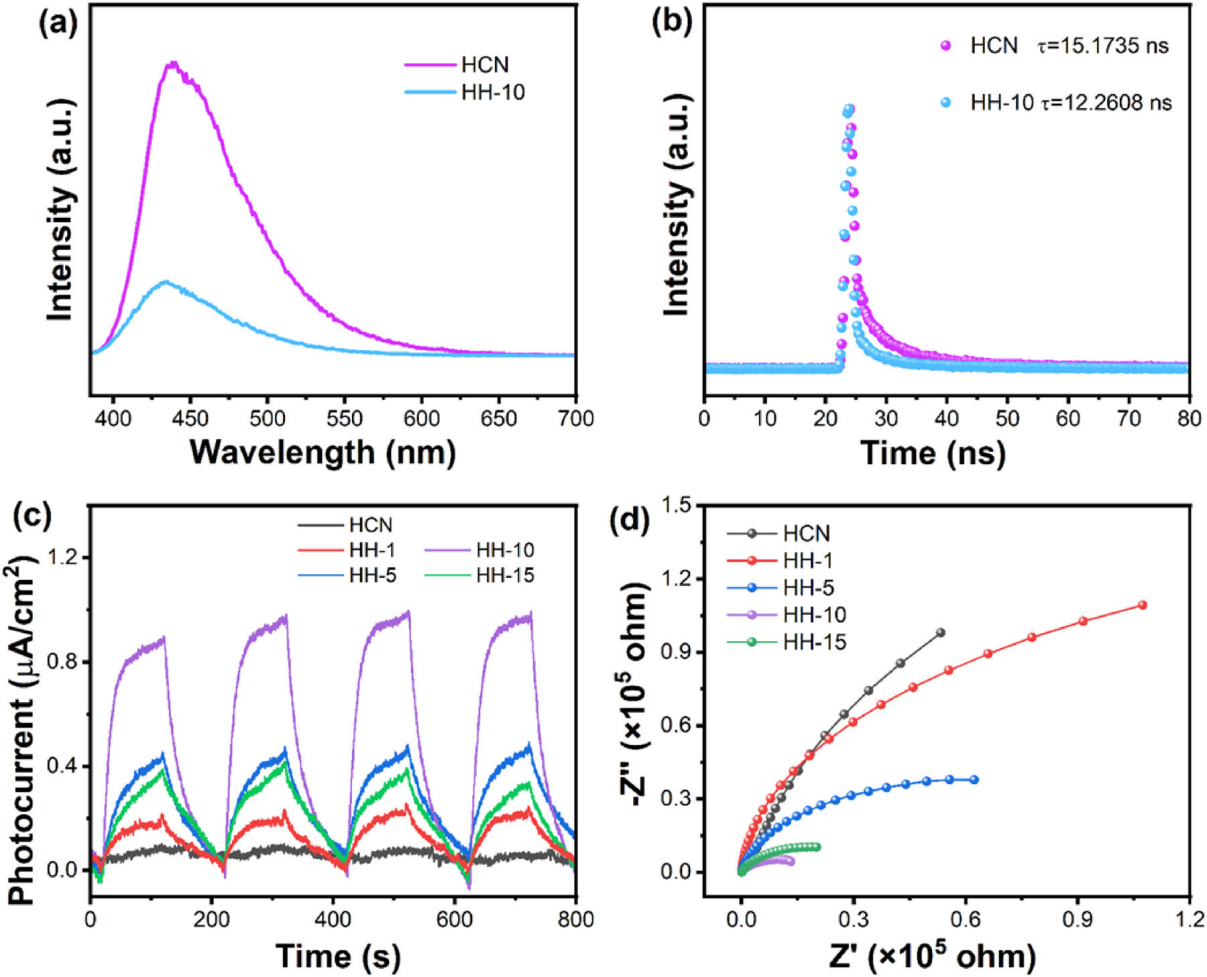
(a) Stable and (b) time‐resolved PL spectra of HCN and HH‐10 composites; (c) transient photocurrent response and (d) electrochemical impedance spectra (EIS) of HCN and HH‐x (x = 1, 5, 10, 15) composites.

Steady and time‐resolved PL spectra of HCN and HH‐10 composites were systematically analyzed to elucidate the critical role of HEA loading on HCN. As shown in Figure 4a, the HH‐10 composite exhibits significantly attenuated PL emission intensity compared to pristine HCN under steady‐state conditions at 365 nm excitation wavelength. Given that stronger emission intensity typically correlates with higher photogenerated electron‐hole recombination rates, these results suggest that HEA incorporation modifies carrier dynamics by enhancing excitation/transfer processes while suppressing recombination pathways [72]. The time‐resolved PL decay analysis (Figure 4b) quantitatively reveals a shortened average carrier lifetime (τ) of 12.2608 ns for the HH‐10 composite, compared to 15.1735 ns for bare HCN. This lifetime reduction mechanistically indicates that HEA cocatalyst sites actively extract photoelectrons from HCN through interfacial charge transfer, thereby inhibiting radiative recombination while promoting electron migration from HCN to HEA [73]. The combined evidence from steady‐state quenching and lifetime reduction (Figure 4a,b) demonstrates two synergistic effects: (1) The effective suppression of bulk/surface recombination through electron trapping at HEA sites; (2) The enhanced migration of photogenerated electrons to surface‐active sites for hydrogen evolution reactions. These optimized charge transfer processes directly correlate with improved photocatalytic activity observed in hydrogen production performance.

**FIGURE 5:**
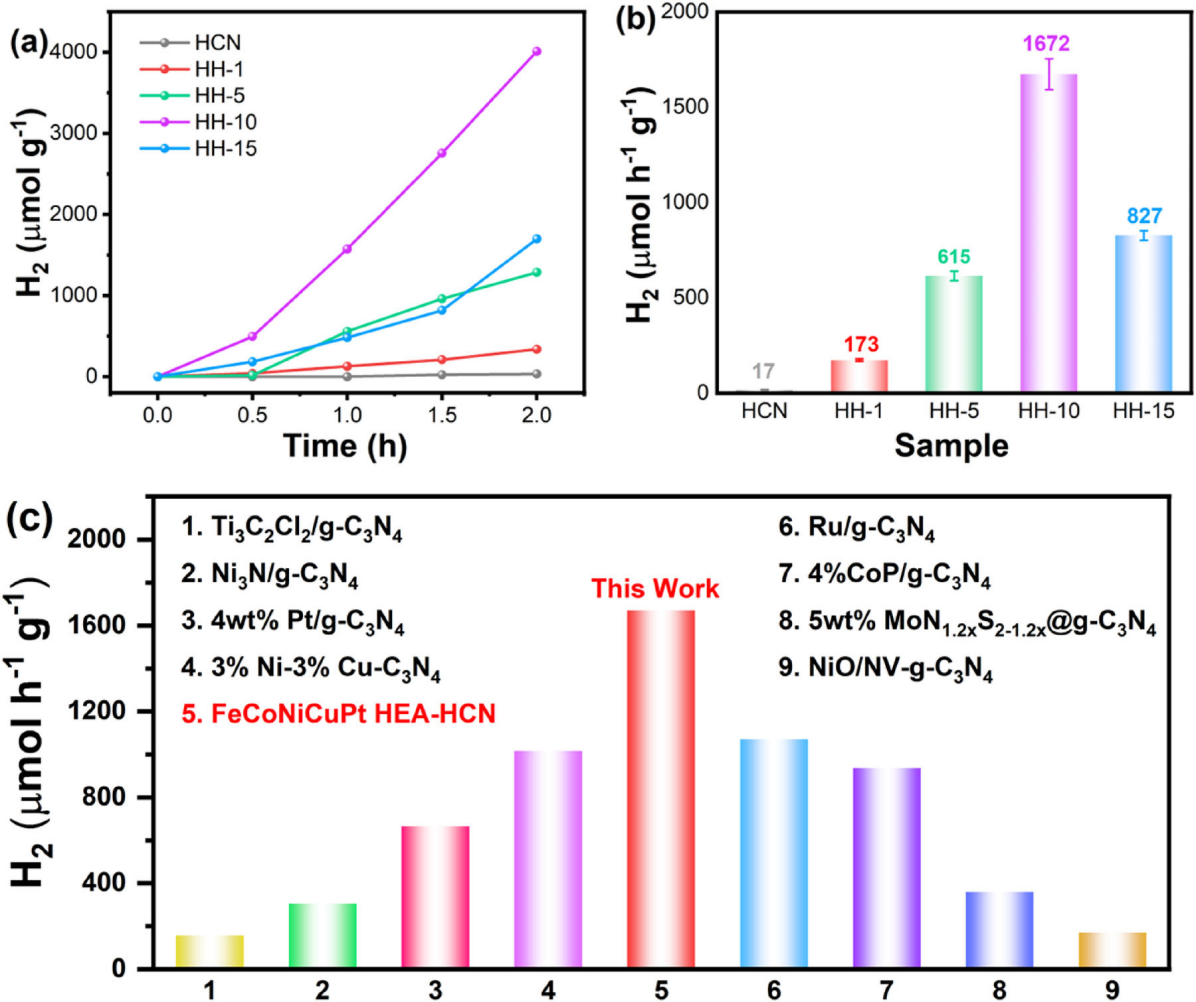
(a,b) Photocatalytic hydrogen precipitation activity and rate of HCN and HH‐x (x = 1, 5, 10, 15) composites; (c) Hydrogen evolution rates for FeCoNiCuPt HEA/HCN in this work compared with representative reported photocatalysts.

To further investigate the critical role of HEA loading on HCN in HH‐x composites and elucidate photogenerated carrier transport/separation dynamics, transient photocurrent response (TPC) and electrochemical impedance spectroscopy (EIS) measurements were conducted. As shown in Figure 4c, HCN exhibits the lowest photocurrent density (0.07 µA/cm^2^), while HEA incorporation progressively enhanced the TPC intensity across the HH‐x series, with HH‐10 demonstrating the strongest photocurrent response (0.96 µA/cm^2^). This trend directly correlates with its superior photocatalytic hydrogen evolution performance (see below, Figure 5a). The enhanced photocurrent density confirms that HEA loading suppresses electron‐hole recombination, improves carrier separation efficiency, and promotes electron transfer kinetics, thereby optimizing utilization of photoexcited charge carriers [48, 74]. EIS analysis (Figure 4d) reveals the charge transfer characteristics of HCN and HH‐x composites. The significantly smaller Nyquist arc radius of HH‐10 compared to HCN indicates the reduced charge transfer resistance [75, 76]. This reduction mechanistically demonstrates that HEA loading on HCN can lower electron migration barriers, facilitate photogenerated carrier separation, and enhance the interfacial charge transfer from HCN to HEA. Collectively, the HH‐10 composite outperforms HCN through synergistic enhancements in light absorption (Figure 3a) and carrier mobility (Figure 4a,b). These improvements enable greater photogenerated carrier density, efficient charge separation/transfer, and abundant active sites, ultimately yielding exceptional photocatalytic hydrogen production performance.

The photocatalytic hydrogen evolution performance of HCN and HH‐x composites with varying HEA loadings is evaluated under AM 1.5G illumination (300 W Xe lamp) using triethanolamine as sacrificial agent, as shown in Figure 5. Pristine HCN exhibits low hydrogen production activity (17 µmol·h^−1^·g^−1^, Figure 5a,b), consistent with its rapid photogenerated carrier recombination observed in optical and photoelectrochemical analyses (Figures 3 and 4). The hydrogen evolution rate increases progressively from 173 to 1672 µmol·h^−1^·g^−1^ as HEA loading rose from 1 to 10 wt.%, demonstrating the significant photocatalytic enhancement achieves through cocatalyst incorporation. However, further increasing HEA loading to 15 wt.% results in reduced activity (Figure 5a), attributing to light shielding effects from excessive cocatalyst coverage that diminished HCN's photon absorption and subsequent carrier generation [5, 77]. The optimal HH‐10 composite demonstrates exceptional performance with a hydrogen production rate of 1672 µmol·h^−1^·g^−1^, representing a 98.35‐fold enhancement over bare HCN (17 µmol·h^−1^·g^−1^).

The cyclic stability and reusability of HH‐10 are evaluated through 10‐h photocatalytic hydrogen evolution tests under simulated sunlight irradiation. As shown in Figure S9a, the hydrogen evolution activity remains stable over five consecutive reaction cycles (10 h total), demonstrating the exceptional operational durability. After‐reaction characterization reveals minimal morphological changes in HH‐10, with SEM images of the cycled catalyst (Figure S10) showing negligible differences compared to fresh samples, confirming robust structural stability. Structural characterization after cycling further reveals the microscopic mechanism underlying the composite's stability. As shown in Figure S11, XRD and XPS analyses indicate that the crystal structure and surface chemical states of the HCN support remained highly stable before and after the reaction, providing a robust skeletal framework for the catalytic system. For the FeCoNiCuPt HEA, its overall chemical composition was preserved, while selective dynamic reconstruction occurred on the surface: the (200) crystallographic plane, with its higher surface energy, preferentially interacted with the environment, forming an ultrathin surface passivation layer that led to a weakening of its XRD diffraction signal. In contrast, the more stable (111) plane was retained. Simultaneously, the Pt 4f XPS peaks exhibited a slight positive shift in binding energy, indicating a minor reduction in the surface electron density of Pt and an adaptive adjustment of its surface electronic state associated with the surface reconstruction. These changes are localized and self‐limiting, not compromising the integrity of the HEA particles. Instead, they likely contribute to maintaining the catalytic activity and structural durability of the composite during long‐term operation through the possible formation of a stable interfacial phase.

Using the identical method, the single‐metal co‐catalyst composite Pt‐HCN and the bimetallic co‐catalyst composite PtNi‐HCN (both with a co‐catalyst loading of 10 wt.%) are further synthesized. Their photocatalytic hydrogen production rates are measured and compared with those of HEA‐HCN and HCN, as shown in Figure S9b. The hydrogen production rate of HEA/HCN is 1672 µmol·h^−1^·g^−1^, which is 98.35 times, 6.12 times, and 1.30 times higher than those of HCN (17 µmol·h^−1^·g^−1^), Pt‐HCN (273 µmol·h^−1^·g^−1^), and PtNi‐HCN (1287 µmol·h^−1^·g^−1^), respectively. This demonstrates the superior performance of the HEA co‐catalyst in this study. Based on the ICP‐OES‐determined composition (Table S1), a semi‐quantitative evaluation of the atomic efficiency of the noble metal Pt is conducted. In the optimal HH‐10 sample, the Pt mass loading is only ∼2.95 wt.%, significantly lower than the 10 wt.% in the Pt‐HCN control. Assuming all Pt atoms are exposed and active, the hydrogen evolution rate per unit mass (or per atom) of Pt in HH‐10 is markedly higher than that of isolated Pt in Pt‐HCN. This preliminary comparison suggests that within the multi‐element matrix of the HEA, a small amount of Pt achieves higher catalytic “atomic economy” through its fusion and electronic interaction with Fe, Co, Ni, and Cu.

More importantly, DFT calculations provide key insights into this “synergistic effect” (Figure 7c). At the HEA/HCN interface, the hydrogen adsorption free energy (ΔG_H*_) for all five metal elements is synergistically optimized to a near‐ideal and closely clustered range (−0.42–−0.69 eV). This indicates that the high‐entropy alloy does not merely provide more physically dispersed sites but creates a unique interfacial microenvironment. This “site ensemble” effect, arising from multi‐element synergy, likely facilitates consecutive steps in complex multi‐step reactions (e.g., adsorption‐desorption in HER), surpassing the adsorption energy optimization limits of single metals (e.g., Pt) or limited elemental combinations (e.g., PtNi). Therefore, the superior performance of the FeCoNiCuPt HEA originates from its optimization of the intrinsic reaction kinetics by reconstructing the catalytic interface through multi‐element synergy, while significantly reducing the precious metal loading. Furthermore, its slightly higher mass activity combined with lower Pt content and better cyclic stability (Figure S9a) highlights its overall potential in terms of cost and durability.

Apparent quantum efficiency (AQE) is one of the important methods to evaluate the catalyst activity. As shown in Figure S12, the AQE of HH‐10 reached 3.23% at 370 nm, but decreased markedly to 0.04% at 456 nm. In contrast, pristine HCN exhibited an AQE of 0.26% at 370 nm and 0.03% at 456 nm. This wavelength‐dependent behavior aligns with the corresponding UV‐vis absorption spectra. Notably, HEA loading significantly enhances the AQE of the HH‐10 composite relative to HCN. The higher AQE at 370 nm indicates efficient generation of photogenerated electron‐hole pairs with suppressed carrier recombination at this wavelength. However, as the wavelength increases to 456 nm, the reduced photon energy substantially diminished charge carrier generation efficiency. Concurrent attenuation of light absorption capacity and exacerbated carrier recombination are identified as primary factors responsible for the drastic AQE decline. Compared with the performance of previously reported g‐C_3_N_4_‐based photocatalytic systems (Figure 5c; Table S2). The hydrogen evolution rate of HEA/HCN composite demonstrates superior photocatalytic performance relative to other photocatalysts. Due to differences in experimental conditions (notably light intensity and sacrificial agent concentration) among the cited literature, the absolute hydrogen evolution rates presented in the table should not be directly compared in a strict quantitative manner. Instead, they serve as a trend indicator for assessing the relative activity levels of the catalysts. The improved photocatalytic performance arises from two synergistic mechanisms: (1) the lamellar architecture of HCN enhances light absorption by increasing specific surface area; (2) the self‐assembled HEA cocatalyst suppresses carrier recombination, reduces charge transfer resistance, and promotes rapid migration of photogenerated carriers to abundant active sites, thus accelerating surface hydrogen evolution reactions. These results confirm that rational cocatalyst engineering effectively balances light utilization, charge dynamics, and surface reactivity for sustainable photocatalytic hydrogen production.

**FIGURE 6:**
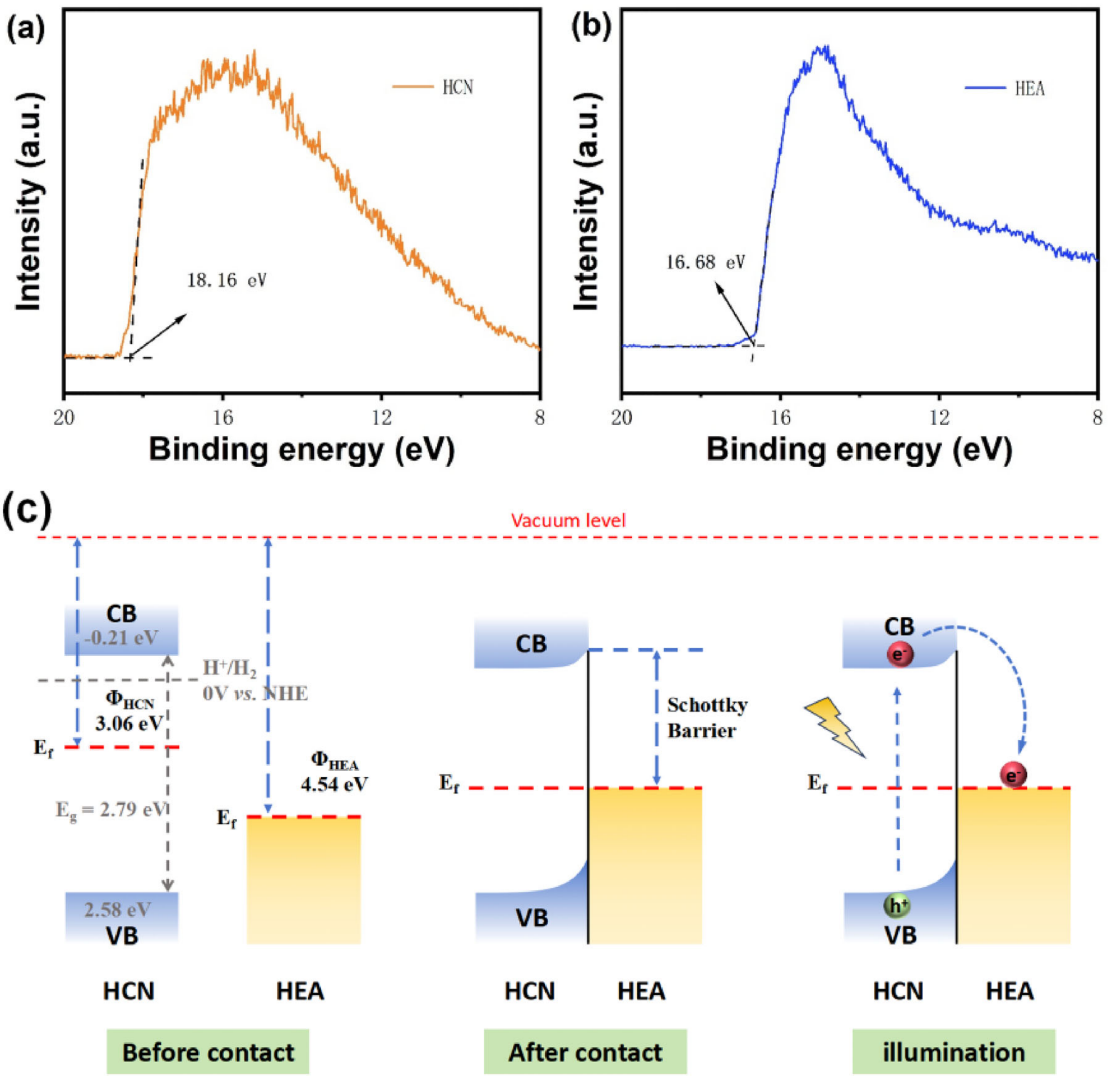
UPS spectra of (a) HCN and (b) HEA; (c) Energy band structure and possible photocatalytic charge transfer mechanisms of HCN/HEA before contact, after contact and illumination.

Ultraviolet photoelectron spectroscopy (UPS) measurements were performed on HCN and HEA to determine their work functions, as shown in Figure 6a,b. The secondary electron cutoff edge analysis revealed work function values of 3.06 eV for HCN and 4.54 eV for HEA [78]. The higher work function of HEA compared to HCN enables the formation of a Schottky junction at their interface during self‐assembly [78, 79, 80]. As illustrated in Figure 6c, when HEA and HCN come into contact, the Fermi level difference drives electron transfer from HCN to HEA until equilibrium is reached. This charge redistribution induces upward band bending in HCN, establishing interfacial Schottky barrier formation [81]. Upon photoexcitation, charge carriers are excited from the valence band (VB) to the conduction band (CB) of HCN. The built‐in electric field facilitates electron migration across the Schottky barrier from HCN to HEA. Importantly, the Schottky barrier effectively prevents captured electrons in HEA from returning to HCN, thereby suppressing charge recombination, promoting spatial separation of photogenerated carriers, prolonging their lifetimes, and ultimately enhancing photocatalytic activity.

**FIGURE 7:**
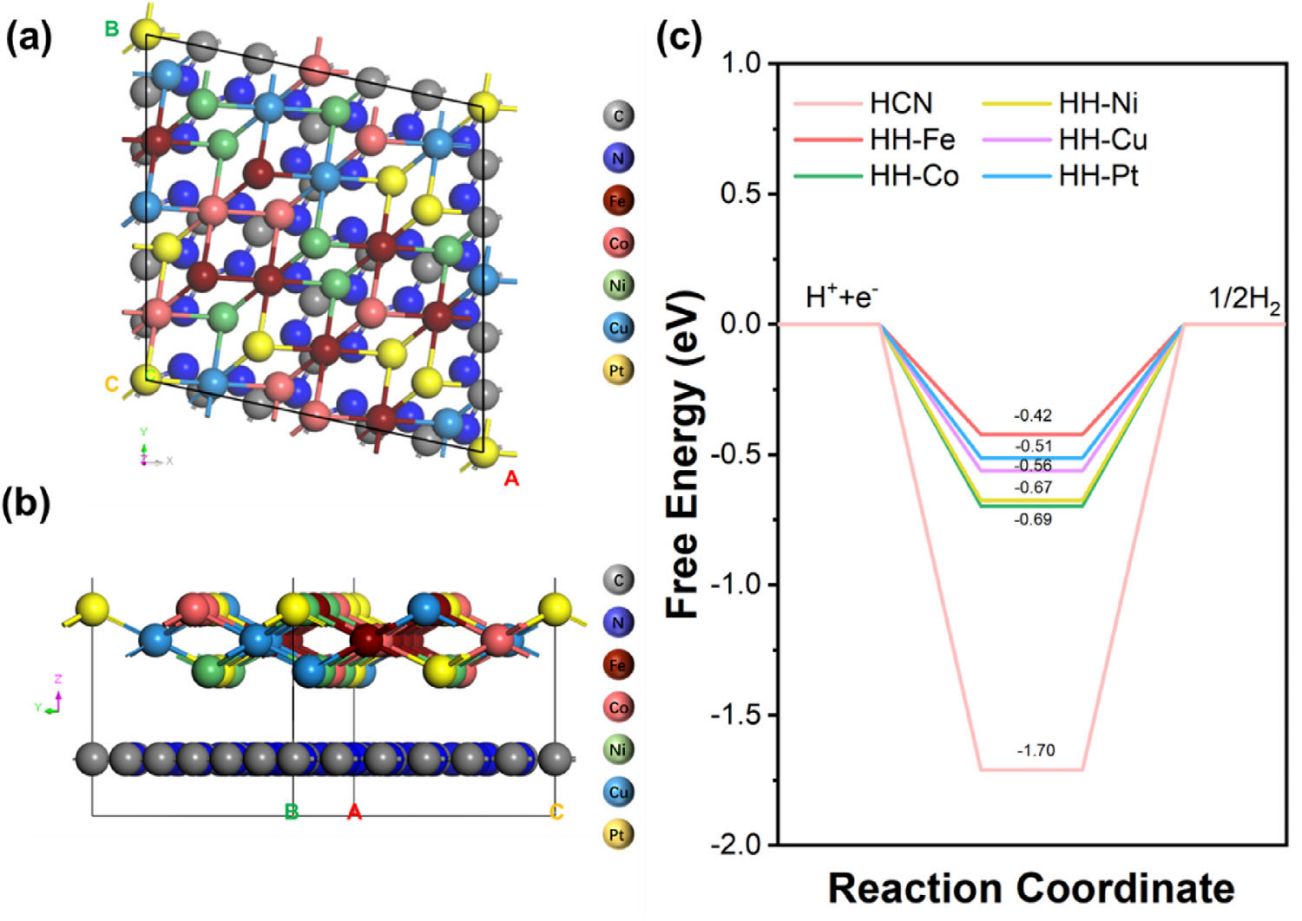
(a) Top‐view structural model, (b) side‐view structural model, and (c) Gibbs free energy profiles of the FeCoNiCuPt HEA/HCN composite.

DFT theoretical simulations were further conducted to investigate the HER at the interface of the photocatalyst. Based on the (111) crystallographic plane identified by transmission electron microscopy (TEM), a slice was taken to construct an atomistic heterostructure of the FeCoNiCuPt high‐entropy alloy (HEA) (111) interfaced with hydrogen cyanide (HCN). The top‐view and side‐view configurations are presented in Figure 7a,b, while alternative side‐view structural models are provided in Figure S13a,b. Charge density difference analysis, widely employed to evaluate interfacial electron transfer, reveals distinct regions of electron depletion (green isosurfaces) and accumulation (yellow isosurfaces), as illustrated in Figure S13c. Notably, although discernible deviations exist between the optimized structural models and theoretical predictions, the charge redistribution trend remains evident: C and N atoms predominantly donate electrons, whereas most Fe, Co, Ni, Cu, and Pt atoms accept electrons at the hybrid interface. Gibbs free energy (ΔG_H*_) serves as a critical descriptor of hydrogen evolution reaction (HER) kinetics. Generally, a ΔG_H*_ value closer to zero facilitates the equilibrium between hydrogen adsorption and desorption, enabling catalytic active sites to rapidly release H_2_ and achieve efficient hydrogen production. As shown in Figure 7c, the ΔG_H*_ value for HCN is ‐1.70 eV, indicating strong hydrogen adsorption but unfavorable desorption of the intermediate H*. From a kinetic perspective, a more negative ΔG_H*_ promotes selective H adsorption. However, thermodynamically, higher energy is required for H desorption, thereby suppressing HER activity [5, 82]. In contrast, the HEA/HCN composite exhibits significantly optimized ΔG_H*_ values at Fe, Co, Ni, Cu, and Pt sites, with values reduced to −0.42, −0.69, −0.67, −0.56, and −0.51 eV, respectively. These results demonstrate that the composite provides more favorable active sites for HER, as the adjusted ΔG_H*_ values approach the ideal range (≈0 eV), thereby enhancing H_2_ desorption efficiency and overall catalytic performance. Among them, the Fe site exhibits a ΔG_H*_ value of −0.42 eV, which is closest to the ideal thermodynamic value of 0 eV, as reflected by its most balanced hydrogen adsorption/desorption capability. Meanwhile, the Pt site, with an optimized ΔG_H*_ of −0.51 eV (the second closest to Fe), remains highly competitive and aligns with its well‐established role as an excellent HER catalyst. These results imply that Fe and Pt are likely the most favorable active sites for the photocatalytic reaction [83, 84, 85]. Collectively, these findings highlight the crucial role of the Fe‐ and Pt‐enriched HEA surface in prolonging charge carrier lifetime and enhancing photocatalytic efficiency.

**SCHEME 2:**
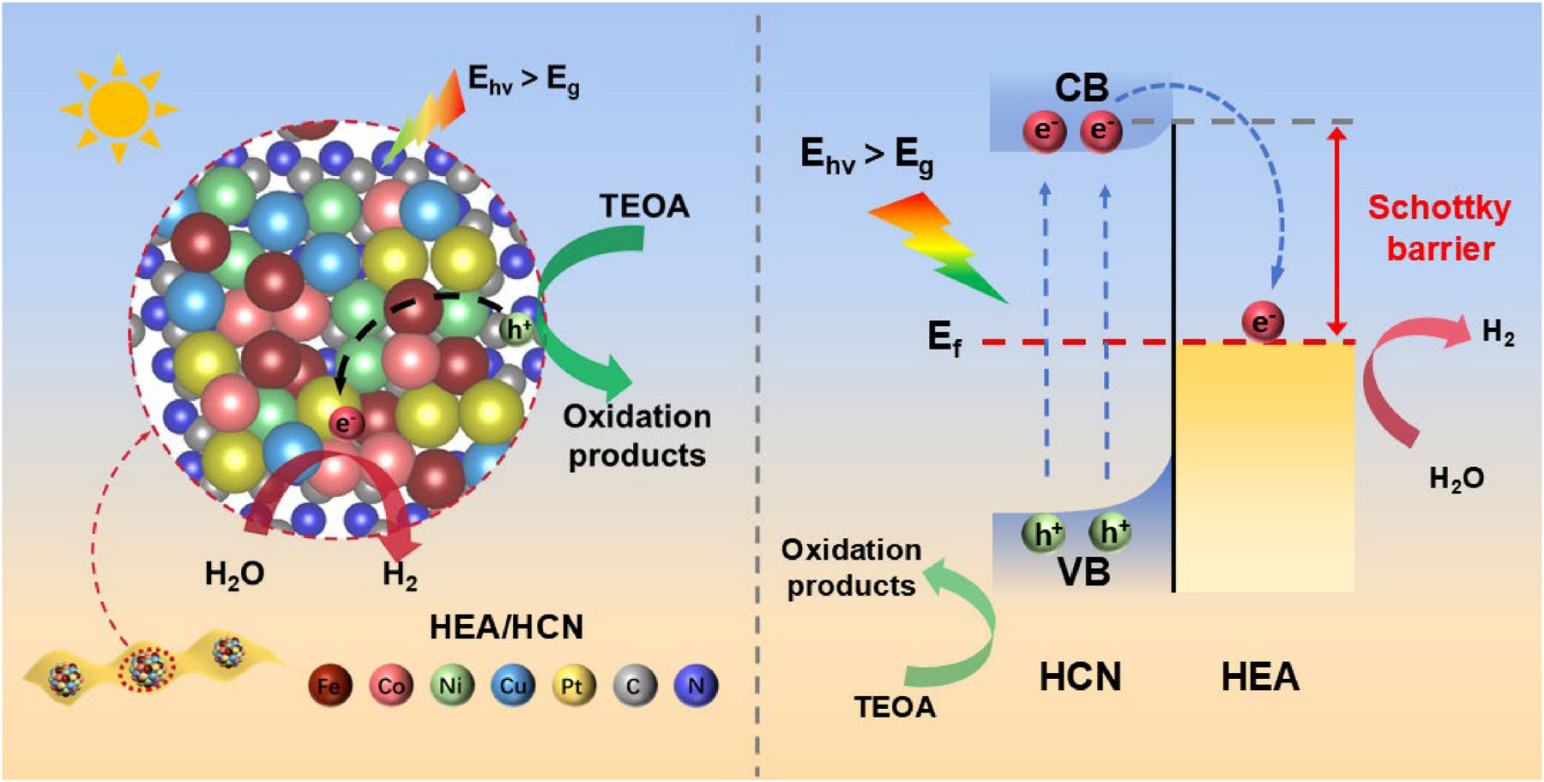
Schematic diagram of the photocatalytic hydrogen production mechanism of HEA/HCN.

Based on the comprehensive analysis, the photocatalytic mechanism of the HEA/HCN composite is proposed in Scheme 2. The incorporation of HEA cocatalyst on HCN enhances the photogenerated carrier concentration under illumination, reduces electron migration resistance, and improves charge transport efficiency. Under light irradiation, photoexcited carriers are generated in HCN, with electrons transitioning from the valence band (VB) to the conduction band (CB). These electrons subsequently migrate to the catalyst surface and are captured by HEA particles, where they participate in the reduction of H^+^ ions from water to produce H_2_. Simultaneously, the residual holes in the VB are consumed through oxidation reactions with the sacrificial agent (triethanolamine, TEOA). The spatial separation of carriers effectively suppresses electron‐hole recombination, significantly enhancing the photocatalytic hydrogen evolution performance. The interfacial Schottky barrier formed between HEA and HCN plays a dual role: it accelerates electron transfer from HCN to HEA via the built‐in electric field, while preventing electron backflow from HEA to HCN. The mechanism involves three key steps: First, the formation of a Schottky junction between the HEA and HCN enables the HEA to function as an efficient electron captor. Subsequently, the injected electrons are rapidly dispersed across its internal multi‐element cooperative network. Finally, a “synergistic active zone”‐consisting of metal sites like Fe and Pt with optimal and closely matched ΔG_H*_ values‐establishes an energetically and kinetically optimized landscape for the complete hydrogen evolution reaction cycle. This synergistic mechanism achieves three critical improvements: (1) effective suppression of carrier recombination, (2) prolonged carrier lifetime, and (3) increased availability of active surface sites. Consequently, the optimized charge dynamics and interfacial engineering collectively enhance the photocatalytic performance of the HEA/HCN composite.

## 3 Conclusion

In summary, HEA/HCN composites are successfully fabricated through electrostatic self‐assembly of high‐entropy alloy (HEA) cocatalysts onto ultrathin protonated g‐C_3_N_4_ nanosheets (HCN NSs) to enhance photocatalytic hydrogen production. Protonation treatment enriches the surface of g‐C_3_N_4_ nanosheets with abundant active sites and enhances their interfacial charge separation capability. The formation of a Schottky heterojunction at the HEA/HCN interface promotes spatial separation of photogenerated electron‐hole pairs, significantly improving photocatalytic activity. Among the synthesized composites, the optimum HEA/HCN composite demonstrates optimal performance with a hydrogen evolution rate of 1672 µmol·h^−1^·g^−1^, representing a 98.35‐fold enhancement compared to pristine HCN. The apparent quantum efficiency (AQE) of HEA/HCN composite reaches 3.23% at λ = 370 nm, further confirming its superior light utilization capability. Mechanistically, the enhanced performance originates from synergistic interfacial interactions between the HEA cocatalyst and HCN substrate. The chemical interaction between HEA and HCN primarily manifests as a physical–electronic coupling mechanism, initiated by electrostatic attraction and dominated by interfacial electron transfer driven by the work function difference. Although this interaction does not involve the formation of traditional strong chemical bonds, it significantly enhances the photocatalytic hydrogen evolution activity by establishing a Schottky junction, optimizing interfacial charge separation efficiency, and modulating the surface hydrogen adsorption free energy. Comprehensive characterization and photoelectrochemical analyses revealed that HEA loading provides abundant reactive centers while optimizing charge dynamics through three key effects: facilitating electron‐hole separation, accelerating interfacial charge transfer, and suppressing carrier recombination. DFT calculations indicate that Fe and Pt are likely the most favorable active sites in the photocatalytic reaction. This work establishes a novel cocatalyst strategy using high‐entropy alloys to boost photocatalytic hydrogen evolution, highlighting the promise of HEA‐based architectures in solar‐to‐energy transformation. This work employs a solvothermal synthesis coupled with electrostatic self‐assembly to fabricate high‐entropy alloy composite catalysts, a method characterized by its mild reaction conditions and simple operational steps, making it a viable candidate for large‐scale preparation. Furthermore, the successful implementation of high‐entropy alloys as multifunctional cocatalysts offers new design principles for advanced photocatalytic materials. More importantly, the inherent compositional tunability and pronounced synergistic effects of high‐entropy alloys (HEAs) offer immense design freedom for developing platform‐type catalytic materials. Building upon the mechanisms of interfacial charge separation and optimized hydrogen adsorption energy revealed in this work, the rational adjustment of HEA composition suggests that these composite architectures can be extended to other critical photocatalytic energy conversion reactions. These include CO_2_ reduction, hydrogen production via plastic waste reforming, and the synthesis of high‐value chemicals. This provides a new design perspective for next‐generation, efficient, stable, and cost‐effective multifunctional solar catalytic systems.

## Conflicts of Interest

The authors declare no conflicts of interest.

## Supporting information




**Supporting File**: advs74691‐sup‐0001‐SuppMat.docx.

## Data Availability

The data that support the findings of this study are available from the corresponding author upon reasonable request.
