# Supplementary material for: FeCoNiCuPt High‐Entropy Alloy Boosts Photocatalytic Hydrogen Production on Protonated Graphitic Carbon Nitride
Source: Adv Sci (Weinh). 2026 Mar 2;13(27):e74691. doi: 10.1002/advs.74691 (PMC13170257; doi:10.1002/advs.74691)
Supplement: Supplementary file 1 — Supporting File: advs74691‐sup‐0001‐SuppMat.docx. [file ADVS-13-e74691-s001.docx]

**Supporting Information**

**FeCoNiCuPt High-Entropy Alloy Boosts Photocatalytic Hydrogen Production on Protonated Graphitic Carbon Nitride**

*Yunzhu Zang, Jiali Ren, Yanjun Xue, Jian Tian^*^*

*State Key Laboratory of Disaster Prevention and Ecology Protection in Open-pit Coal Mines, Shandong Key Laboratory of Special Epoxy Resin, School of Materials Science and Engineering, Shandong University of Science and Technology, Qingdao 266590, China. Email: jiantian@sdust.edu.cn (J. Tian)*

**Experimental Section**

**Materials**

Urea (CH_4_N_2_O, 99%), iron(III) acetylacetonate (99%), cobalt(II) acetylacetonate (97%), nickel(II) acetylacetonate (95%), copper(II) acetylacetonate (97%), platinum(II) acetylacetonate (97%) and cyclohexane (C_6_H_12_, 99.7%) were bought from Macklin. Ethanol (C_2_H_5_OH, ≥ 99.0%) and triethanolamine (TEOA, ≥ 98.0%) were bought from Greagent. Hydrochloric acid (HCl, 37wt.%) and acetone (CH_3_COCH_3_, ≥ 99.5%) were bought from [ChengDu Chron Chemicals Co.,Ltd](http://www.chronchem.com/en/). The deionized water was used for all experiments and all chemicals were used directly without further purification.

**Preparation of g-C_3_N_4_ nanosheets (NSs)**

Lamellar g-C_3_N_4_ was obtained following one-step thermal polycondensation under ambient atmospheric conditions. Specifically, 20 g of urea was weighed into an alumina crucible, covered with a lid, and subjected to a thermal treatment protocol in a muffle furnace. The furnace temperature was raised to 550°C at a heating rate of 5°C min^-1^, followed by a 2-hour isothermal calcination step, ultimately yielding g-C_3_N_4_ nanosheets (NSs).

**Preparation of protonating g-C_3_N_4_ nanosheets (HCN)**

Protonated g-C_3_N_4_ (HCN) was prepared through acid treatment as follows: 2 g of g-C_3_N_4_ nanosheets (NSs) were dispersed in 20 mL of concentrated hydrochloric acid (37 wt.%), magnetically stirred for 12 h at room temperature, and then subjected to centrifugal washing with deionized water until the supernatant reached neutral pH (pH = 7). The obtained precipitate underwent thermal drying at 60°C overnight to obtain the HCN product.

**Preparation of FeCoNiCuPt high-entropy alloys (HEA)**

First, five metal acetylacetonates (each 0.1 mmol) were weighed to prepare the precursor solution, and then were dissolved in a mixture of acetone-ethanol (50:50 vol%). The total metal concentration of all metal precursors (acetylacetonate) was 5 × 10^-3^ M. The above solution was then transferred to a 100mL PTFE-sealed hydrothermal reaction vessel and heated at 200°C for 4 h. Upon reaching room temperature through spontaneous cooling, the dark-colored material was isolated via centrifugal separation and washed twice with acetone, and then dried under vacuum at 60°C to obtain FeCoNiCuPt high-entropy alloys (HEA).

**Preparation of HEA/HCN composites**

The HEA/HCN composites were synthesized by electrostatic self-assembly method. 200 mg of HCN was ultrasonically dispersed uniformly in 40 mL of anhydrous ethanol, and 20 mg of HEA was dispersed in 10 mL of cyclohexane, and then added dropwise to the HCN solution. After sonication for about 5 h, the obtained solid sample was harvested through centrifugation and rinsed with anhydrous ethanol, dried under vacuum at 60 °C, and the prepared composite was named HH-10. Similarly, by adjusting the amount of HEA, a series of composites containing different amounts of HEA, named HH-x (x = 1, 5, 10, 15), were obtained, where x is the weight percentage of HEA in the composite.

**Characterization**

Phase analysis of the powdered specimens was conducted using a Rigaku D/Max 2500 PC X-ray diffraction system (XRD). Fourier transform infrared (FT-IR) spectra were recorded on a Nicolet iS10 spectrometer. The nanostructure and surface morphology of the samples were tested employing field emission scanning electron microscopy (FESEM, Apreo S HiVac, USA) and high-resolution transmission electron microscopy (HRTEM, Talos F200X, USA) combined with energy dispersive x-ray energy spectroscopy (EDX). Elemental analysis was performed using inductively coupled plasma optical emission spectrometry (ICP-OES) with an Agilent 725 ES instrument. X-ray photoelectron spectroscopy (XPS) was performed on the samples using a Thermo ESCALAB 250XI instrument. Photoluminescence (PL) spectra were performed on a FLS980 fluorescence spectrometer (Edinburgh Instruments, UK). The UV-Vis diffuse reflectance spectra (DRS) of the samples were determined using a Hitachi UV-2550 UV-Vis spectrophotometer. UPS testing was conducted using the Kratos AXIS Supr. Zeta potentials were measured using a Zetasizer Nano ZS90 analyzer (Malvern, UK).

**Photoelectrochemical test**

The photoelectrochemical tests were performed using a 300 W xenon lamp as the light source. Electrochemical measurements including transient photocurrent, EIS, and Mott-Schottky analysis were conducted on a Chenhua CHI660D workstation (Shanghai, China) using a conventional three-electrode system with the electrolyte comprising 0.5 M Na₂SO₄, the synthesized catalytic materials as the working electrode, the Pt wires as the counter electrode, and Ag/AgCl as the reference electrode.

**Photocatalytic hydrogen production test**

In a 50 mL system of 20 vol% TEOA aqueous solution, 20 mg of catalyst was added and stirred and dispersed, and photocatalytic hydrogen production was tested in a 100 mL photoreactor. An AM-1.5 filter 300 w xenon lamp was used as the irradiation source. The released gas was detected using a Techcomp GC-7920 gas chromatography system equipped with thermal conductive detection (TCD). The apparent quantum efficiency (AQE) of the catalyst was tested under the same photocatalytic conditions and was calculated as follows:

$N=\frac{Pt\lambda}{hc}=\frac{EA_{R}t\lambda}{hc}$ (1)

$\text{AQE=}\frac{\text{number of evolved }\text{ H}_{\text{2}}\text{ molecules ×2}}{\text{number of incident photons}}\times100\%$ (2)

*N* denotes the number of incident photons, calculated based on the photon flux. *P* represents the power density. *t* is the irradiation time. *E* signifies the average irradiance. *A_R_* refers to the illuminated area. *λ* stands for the incident wavelength. *h* is Planck's constant, and *c* is the speed of light in vacuum.


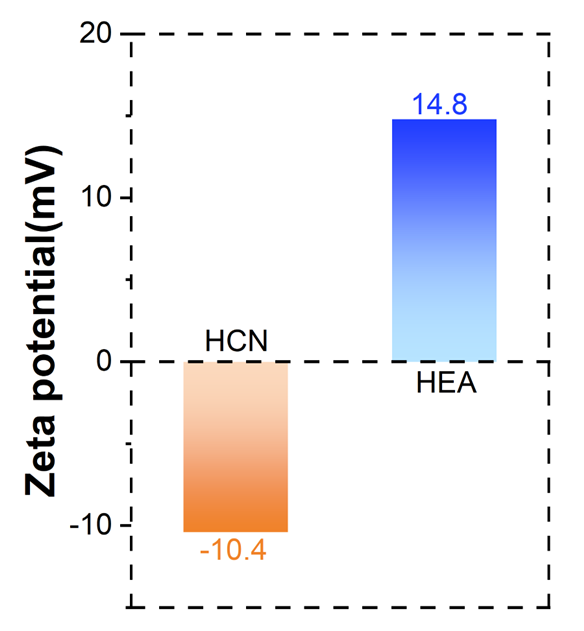


**Figure S1.** Zeta potentials of HEA and HCN.


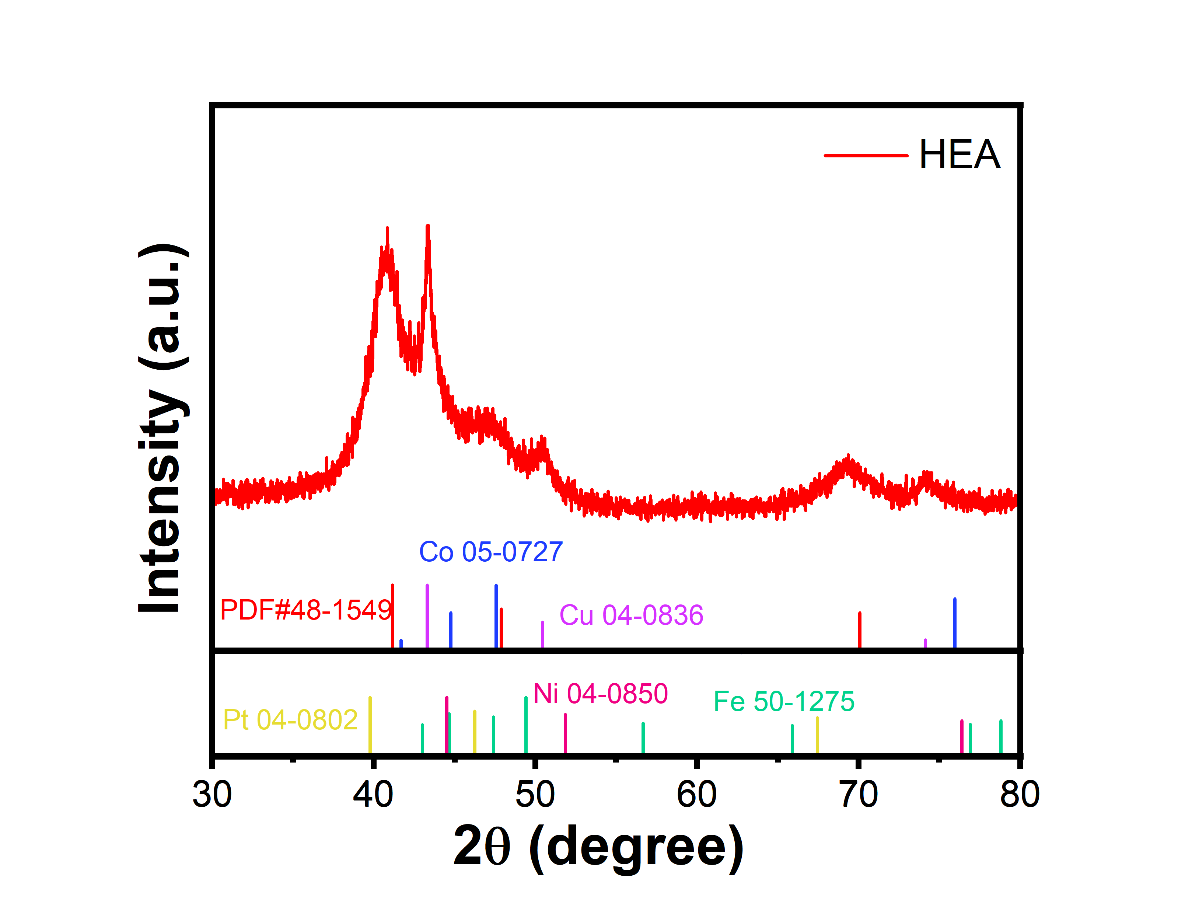


**Figure S2.** XRD spectrum of HEA.


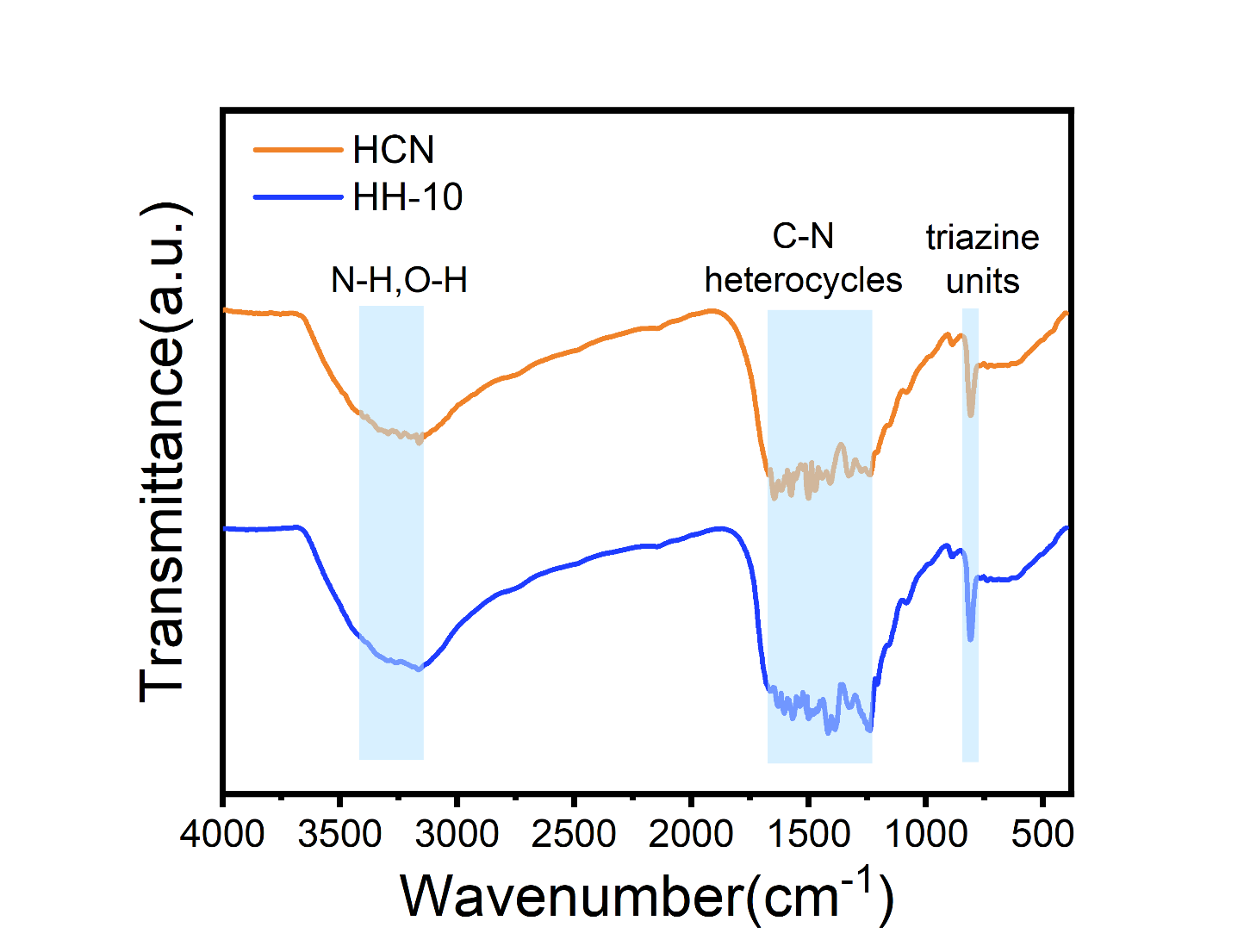


**Figure S3.** FT-IR spectra of HCN and HH-10.

**Table S1.** ICP-OES test results of HEA and HH-10.

| Sample | Fe (wt.%) | Co (wt.%) | Ni (wt.%) | Cu (wt.%) | Pt (wt.%) |
| --- | --- | --- | --- | --- | --- |
| HEA | 12.376 | 15.294 | 15.376 | 19.459 | 30.094 |
| HH-10 | 1.403 | 1.567 | 1.623 | 1.861 | 2.946 |


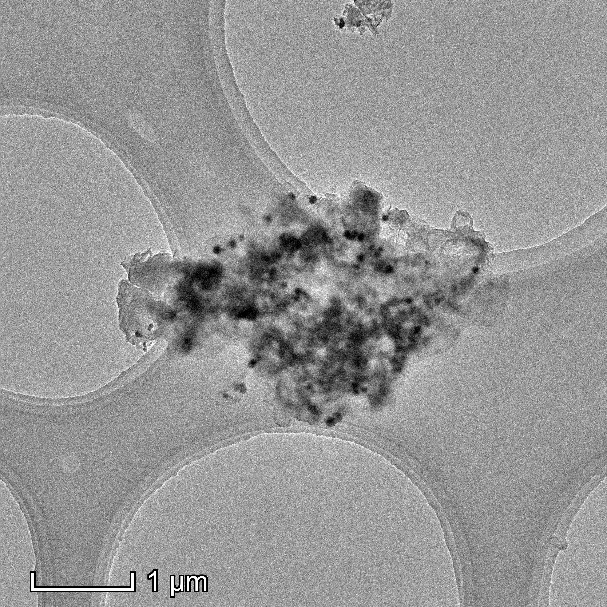


**Figure S4.** TEM image of HH-10 composite.


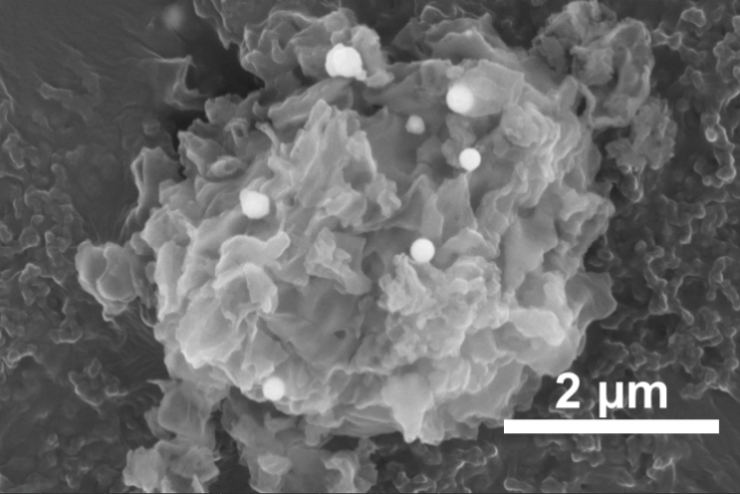


**Figure S5.** SEM image of the HH-10 composite.

**
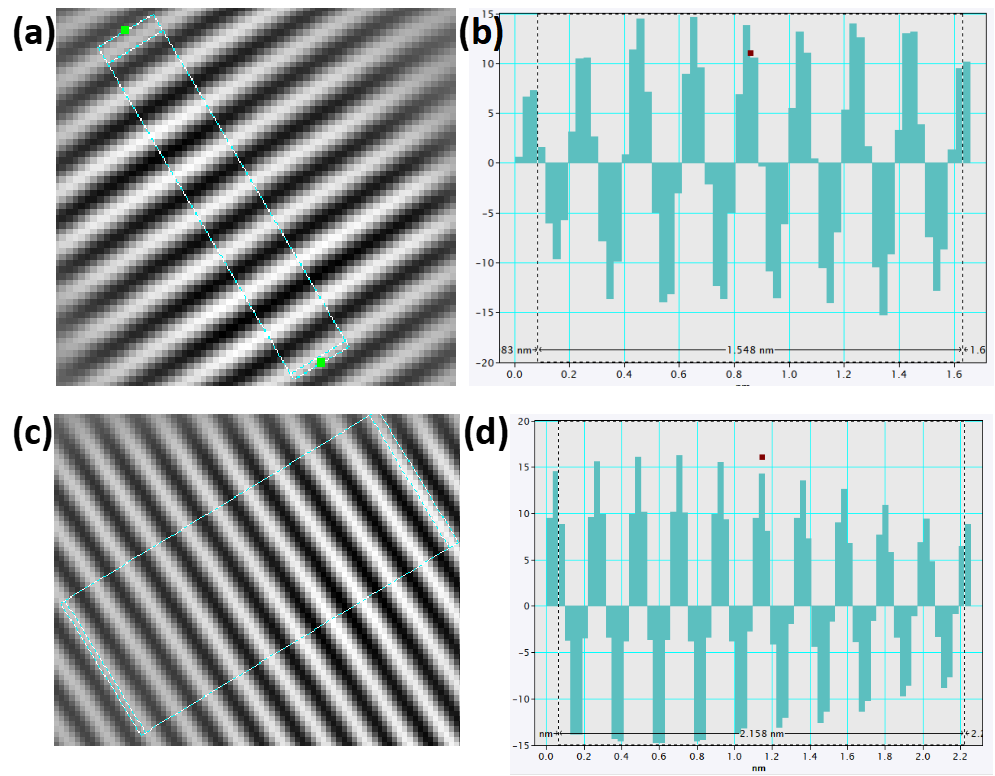
**

**Figure S6.** (a) FFT pattern and (b) lattice spacing profile of the (200) plane; (c) FFT pattern and (d) lattice spacing profile of the (111) plane.

**
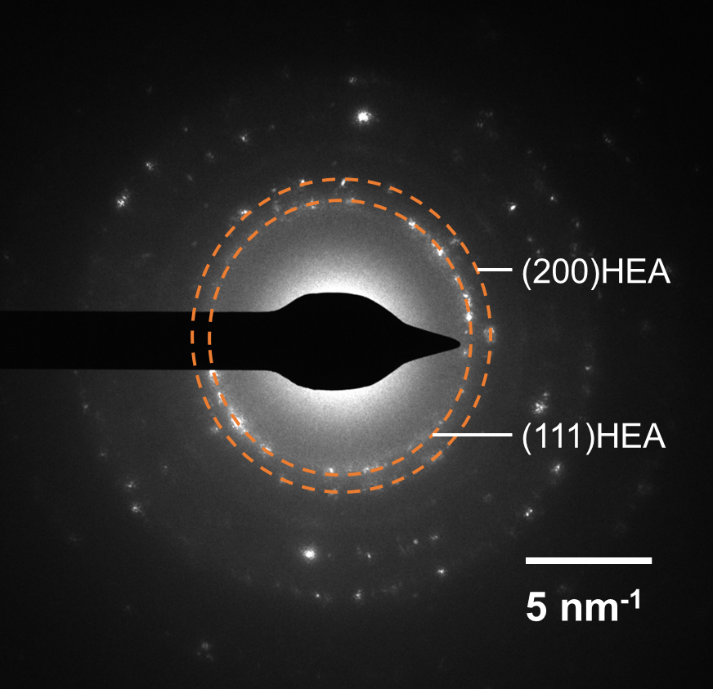
**

**Figure S7.** SAED pattern of HH-10 composite.

**
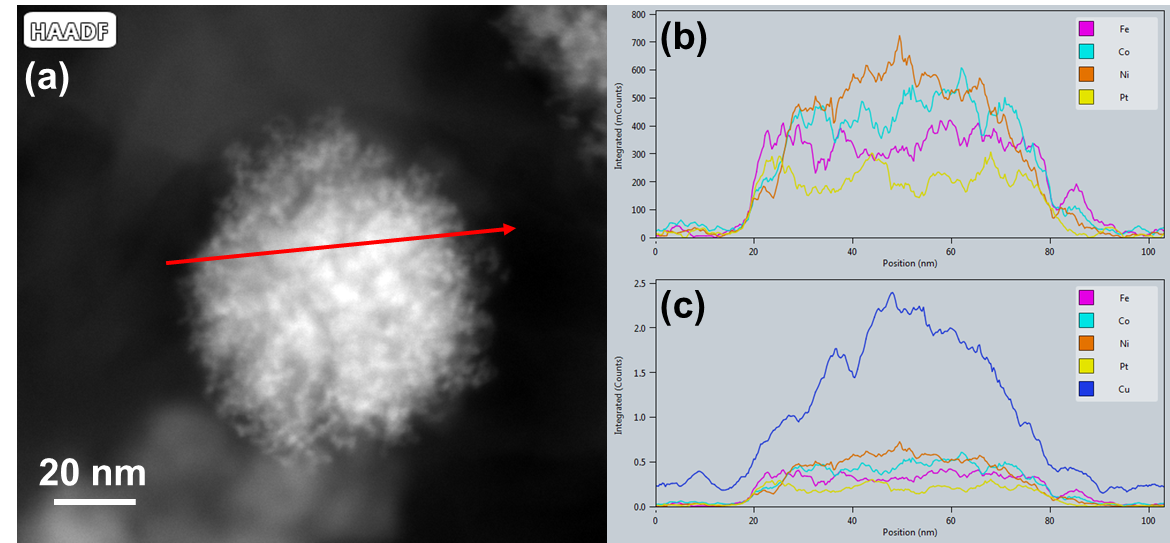
**

**Figure S8.** (a-c) HAADF-STEM image of the HH-10 composite with elemental line-scan profiles.


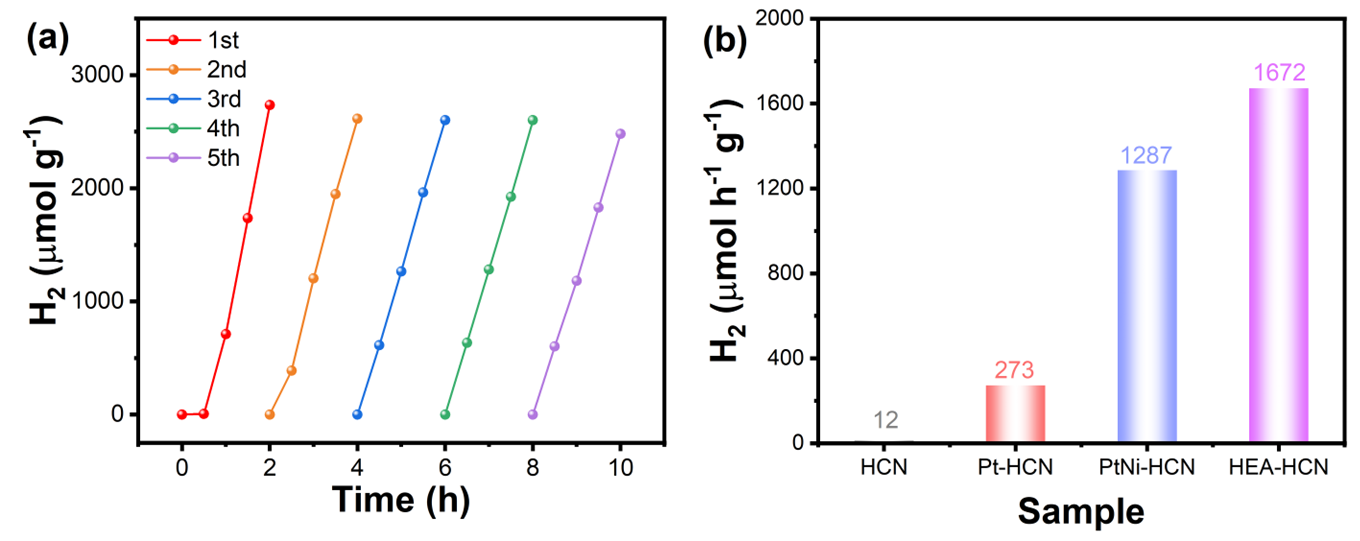


**Figure S9.** (a) Cyclic stability test for photocatalytic hydrogen production of HH-10 composites; (b) Photocatalytic hydrogen evolution rates of HCN, Pt-HCN, PtNi-HCN, and HEA/HCN composites.


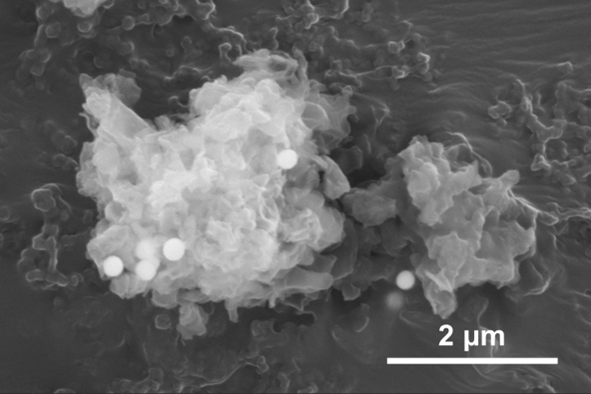


**Figure S10.** Scanning electron microscope image of HH-10 after cyclic stability experiments.


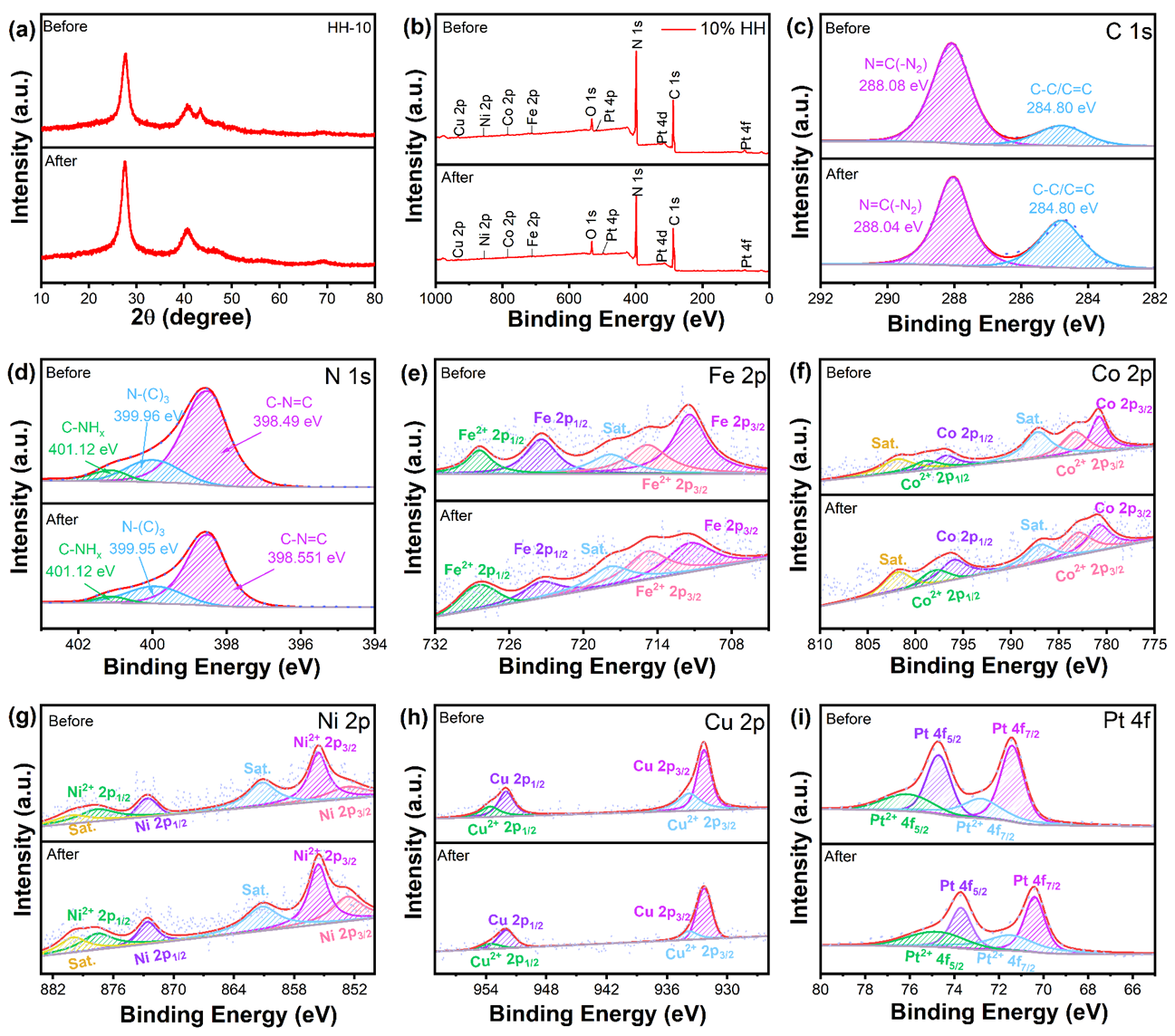


**Figure S11.** (a) XRD patterns of the HH-10 composite before and after cycling; (b) Survey, (c) C 1s, (d) N 1s, (e) Fe 2p, (f) Co 2p, (g) Ni 2p, (h) Cu 2p and (i) Pt 4f XPS spectra of HH-10 before and after cycling.


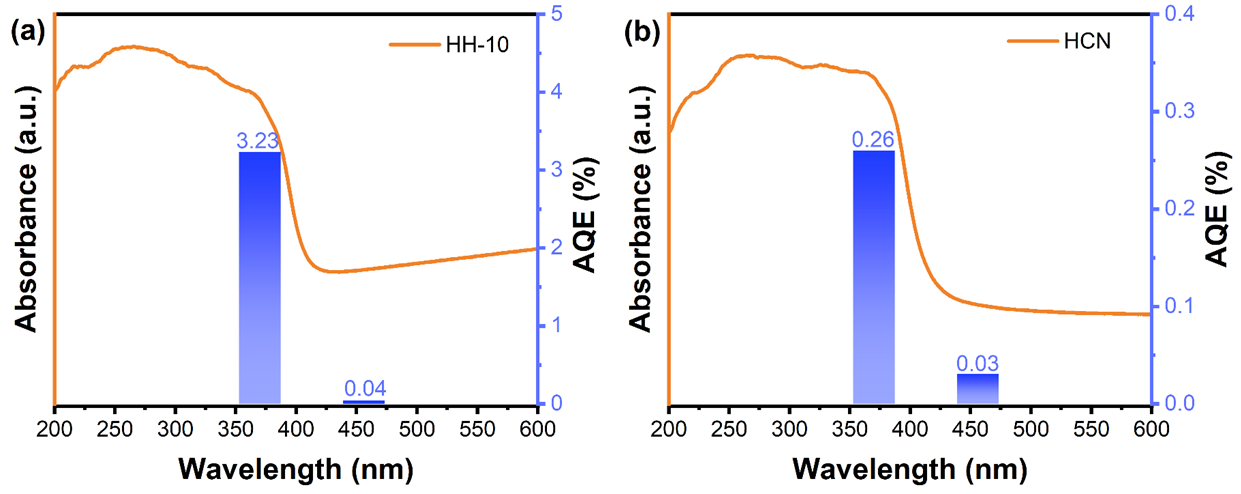


**Figure S12.** Wavelength-dependent apparent quantum efficiency (AQE) values of (a) HH-10 and (b) HCN.

**Table S2.** Comparison of hydrogen evolution rate between FeCoNiCuPt HEA-HCN and other photocatalysts.

| **Catalyst** | **Test conditions** | **Light source** | **H_2_ evolution**  **(μmol·h^-1^·g^-1^)** | **Ref.** |
| --- | --- | --- | --- | --- |
| NiO/NV-g-C_3_N_4_ | 10 vol% TEOA aqueous solution | 3 W LED lamp, λ = 420 nm | 170.6 | [1] |
| Ru/g-C_3_N_4_ | 10 vol% TEOA aqueous solution | 300 W Xe lamp with 400 nm cutoff optical filter | 1070 | [2] |
| 4%CoP/g-C_3_N_4_ | 12.5 vol% TEOA aqueous solution | 350 W Xe lamp | 936 | [3] |
| 4wt% Pt/g-C_3_N_4_ | 12.5 vol% TEOA aqueous solution | 350 W Xe lamp | 665 | [3] |
| Ni_3_N/g-C_3_N_4_ | 20 vol% TEOA aqueous solution | 300 W Xe lamp with a UV cutoff filter λ > 420 nm | 305.4 | [4] |
| 5wt% MoN_1.2x_S_2-1.2x_@g-C_3_N_4_ | 20 vol% TEOA aqueous solution | 300 W simulated solar light source | 360.4 | [5] |
| T_i3_C_2_Cl_2_/g-C_3_N_4_ | 10 vol% TEOA aqueous solution | 300 W Xe lamp λ > 420 nm | 155.9 | [6] |
| 3% Ni-3% Cu-C_3_N_4_ | 10 vol% TEOA aqueous solution | 300 W Xe lamp with AM-1.5G filter | 1017 | [7] |
| 10wt% FeCoNiCuPt HEA/HCN | 20 vol% TEOA aqueous solution | 300 W Xe lamp with AM-1.5G filter | 1672 | This work |


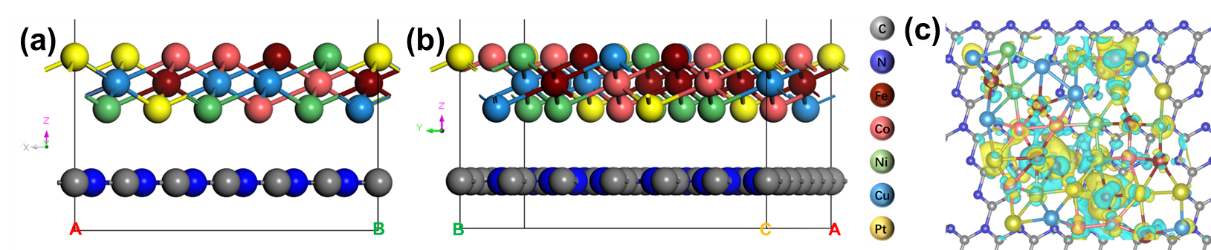


**Figure S13.** (a, b) Side -view structural models and (c) optimized differential charge density of FeCoNiCuPt HEA-HCN composite.

**References**

[1] Y. Liao, J. Yang, G. Wang, J. Wang, K. Wang, S. Yan, *Rare Met.* **2022**, 41, 396-405.

[2] J. Ning, Y. Chen, T. Wang, J. Xiong, *Int. J. Electrochem. Sci.* **2022**, 17, 220855.

[3] K. Qi, W. Lv, I. Khan, S. Liu, *Chin. J. Catal.* **2020**, 41, 114-121.

[4] J. Ge, Y. Liu, D. Jiang, L. Zhang, P. Du, *Chin. J. Catal.* **2019**, 40, 160-167.

[5] X. Wei, M. Wang, S. Ali, J. Wang, Y. Zhou, R. Zuo, Q. Zhong, C. Zhan, *Int. J. Hyd. Energy* **2024**, 89, 691-702.

[6] H. Zhou, J. Tian, R. Wang, D. Zhan, P. Liu, R. Chen, Y. Huang, Z. Liu, C. Han, *Ceram. Int.* **2023**, 49, 13042-13049.

[7] Y. Zhang, J. Yang, Y. Zhang, X. Wang, C. Feng, H. Jin, X. Hou, P. Li, Q. Dong, *Appl. Surf. Sci.* **2025**, 700, 163200.
